# A short pragmatic tool for evaluating community engagement: Partnering for Health Improvement and Research Equity

**DOI:** 10.3389/fpubh.2025.1539864

**Published:** 2025-06-11

**Authors:** John G. Oetzel, Blake Boursaw, Lenora Littledeer, Sarah Kastelic, Page Castro-Reyes, Juan M. Peña, Patricia Rodriguez Espinosa, Shannon Sanchez-Youngman, Lorenda Belone, Nina Wallerstein

**Affiliations:** ^1^School of Management and Marketing, University of Waikato, Hamilton, New Zealand; ^2^College of Nursing, University of New Mexico, Albuquerque, NM, United States; ^3^Regional EEO Office, Indian Health Service, Oklahoma City, OK, United States; ^4^National Indian Child Welfare Association, Portland, OR, United States; ^5^Community-Campus Partnerships for Health, Raleigh, NC, United States; ^6^Department of Psychology, San Jose State University, San Jose, CA, United States; ^7^Department of Epidemiology and Population Health, Stanford University, Palo Alto, CA, United States; ^8^College of Population Health, Center for Participatory Research, University of New Mexico, Albuquerque, NM, United States

**Keywords:** community-based participatory research, community-engaged research, pragmatic measurement, patient-engaged research, CBPR conceptual model

## Abstract

**Background:**

As community-engaged research (CEnR), community-based participatory research (CBPR) and patient-engaged research (PEnR) have become increasingly recognized as valued research approaches in the last several decades, there is need for pragmatic and validated tools to assess effective partnering practices that contribute to health and health equity outcomes. This article reports on the co-creation of an actionable pragmatic survey, shortened from validated metrics of partnership practices and outcomes.

**Methods:**

We pursued a triple aim of preserving content validity, psychometric properties, and importance to stakeholders of items, scales, and constructs from a previously validated measure of CBRP/CEnR processes and outcomes. There were six steps in the methods: (a) established validity and shortening objectives; (b) used a conceptual model to guide decisions; (c) preserved content validity and importance; (d) preserved psychometric properties; (e) justified the selection of items and scales; and (f) validated the short-form version. Twenty-one CBPR/CEnR experts (13 academic and 8 community partners) completed a survey and participated in two focus groups to identify content validity and importance of the original 93 items.

**Results:**

The survey and focus group process resulted in the creation of the 30-item Partnering for Health Improvement and Research Equity (PHIRE) survey. Confirmatory factor analysis and a structural equation model of the original data set resulted in the validation of eight higher-order scales with good internal consistency and structural relationships (TLI > 0.98 and SRMR < 0.02). A reworded version of the PHIRE was administered to an additional sample demonstrating good reliability and construct validity.

**Conclusion:**

This study demonstrates that the PHIRE is a reliable instrument with construct validity compared to the larger version from which it was derived. The PHIRE is a straightforward and easy-to-use tool, for a range of CBPR/CEnR projects, that can provide benefit to partnerships by identifying actionable changes to their partnering practices to reach their desired research and practical outcomes.

## Background

As community-engaged research (CEnR), community-based participatory research (CBPR) and patient-engaged research (PEnR) have become increasingly recognized as valued research approaches in the last several decades, there is need for models and validated tools to assess effective partnering practices that contribute to health and health equity outcomes. While many systematic reviews have identified a range of impacts of engagement practices on outcomes (1–4), the science of creating strong, reliable, and valid measurements has lagged. With increasing National Institutes of Health (NIH) and foundation funding mandates for community-academic partnerships, even more important is having pragmatic instruments that can serve as evaluation or collective reflection opportunities to strengthen partnership capacities to achieve desired outcomes.

Early on, building from the coalition (5), empowerment (6), organizational development and capacity (7), and team science (8) literatures, individual research projects developed their own mixed-methods evaluations, such as the Detroit Urban Research Center (9) or relied on existing tools such as the Wilder Collaboration instrument (10). More recently multiple research projects and task forces have produced validated instruments that come from collaborative research projects with diverse populations, regions, and health issues, with a few systematic reviews (11–13). Noteworthy among these are three national efforts that have created an engagement model and developed or identified validated tools.

The earliest effort, now called Engage for Equity (E^2^), was launched in 2006 with the goal of developing an assessment tool of measures and metrics of partnering processes and outcomes, and of identifying promising or best practices that contribute to health and health equity outcomes (14). In the first funding from the NIH, the University of New Mexico Center for Participatory Research team, with partner organizations and a community-academic “Think Tank” as a national advisory group and much community consultation, first reviewed the literature and created a CBPR conceptual model, composed of four domains: the political-economic and social *contexts* under which partnerships operate; *partnering practices*, including who is involved and how well partners interact in their relationships and their formal agreements; collaborative implementation of *research and intervention actions*; and projected *outcomes*, including intermediate and long-term health and social justice outcomes (15, 16). With the second NIH funding, the national team developed the Community Engagement Survey (CES) with questions from each of the CBPR model domains, conducted internet surveys of academic and community partner teams from 200 federally-funded diverse CBPR projects, and produced a first set of promising practices that contribute to outcomes (17) and psychometrically-validated scales (18). A companion E2 Key Informant Survey for principal investigators asked about the facts of each project. In the third NIH funding, the team refined the CES instrument, translated it into Spanish (19) and surveyed another 210 federally-funded partnerships, enabling refinement of psychometrics (20) and analysis of pathways of which practices contribute to outcomes, with a focus on collective empowerment and shared governance (21, 22). In this third stage, Partnership Data Reports^1^ were created to return their own data to partnered teams who attended E^2^ intervention workshops, based on Paulo Freire’s praxis of promoting collective reflection to strengthen team actions (23, 24). The 93 CES questions were divided into eight higher order constructs, which also facilitated team reflections: partnership capacity, structural governance, commitment to collective empowerment, relationships, community-engagement in research, synergy, intermediate outcomes, and long-term outcomes.

Several related measurement projects are relevant to this work. The NIH-funded Measurement Approaches to Partnership Success (MAPS) project was launched in 2019, by the Detroit Community-Academic Urban Research Center, with the goal of developing and validating an instrument focused on partnership success (25, 26). The MAPS model was developed with a CBPR process through their national advisory board, basing constructs on their conceptual framework of partnership evaluation refined over twenty years. Internet surveys were conducted with 55 successful CBPR partnerships, defined as being over 6 years of existence, typically with multiple funding cycles or projects. The validated questionnaire organized 81 questions into seven dimensions: equity in the partnership, reciprocity, competence enhancement, synergy, sustainability, realization of benefits over time, and achievement of long-term partnership goals/outcomes (27–29).

The “Assessing Meaningful Community Engagement” model is the product of a National Academy of Medicine (NAM) taskforce, which brought together academic and community experts to review the field of CEnR, develop a broader engagement model, and compile a summary of all validated instruments to date (30). The literature review of validated instruments identified 28 instruments that cover constructs within the NAM model, with the E^2^ Community Engagement Survey (CES), one of only three in the nation that covers all NAM domains. The review also included the Key Informant Survey (KIS), the E^2^ companion instrument, and the Spanish translation of the CES and KIS, which are two of only three Spanish surveys (19). A formal guide provides an overview of each instrument, illustrating how each aligns with the model, its psychometrics, and its potential uses (31).

While these instruments have served to identify appropriate constructs of promising or best partnering practices and their impact on outcomes, many of them have high response burden due to the large number of items and are limited in their pragmatic use as partnership evaluation tools. According to Glasgow and Riley (32), pragmatic measures should, at minimum, have the properties of being important to stakeholders, low burden, actionable, and sensitive to change. This paper presents one effort by the UNM-Center for Participatory Research E^2^ team to create a pragmatic reduced-item tool from the CES that can be used by CBPR/CEnR community members, practitioners and researchers as an annual evaluation and planning opportunity. By using a short tool, partners can collectively reflect on their own data, assess their current capacities and identify the areas they want to strengthen over the next year to become more effective at reaching their outcomes.

This paper provides an overview of the measure shortening process from the E^2^ CES to a new shorter tool, called Partnering for Health Improvement and Research Equity (PHIRE). CES higher-order constructs were maintained, and quantitative and qualitative methods were used to reduce items. Discussion includes initiatives which have piloted the PHIRE, its Spanish translation, and importance of cultural and community context. Implications for use as a pragmatic evaluation and collective reflection tool by CBPR/CEnR projects and other community collaborations are presented, with future directions for further translation and validation efforts.

## Methods

The original survey and psychometric properties are discussed elsewhere (20). To develop a pragmatic instrument of CBPR processes and outcomes, we pursued a triple aim of preserving content validity, psychometric properties, and importance to stakeholders of items, scales, and constructs from the E^2^ CES by adapting six steps outlined in Goetz et al. (33). We embed the participants, data collection, and analysis within these six steps as detailed in the following subheadings. The study involved human participants and was reviewed and approved by the University of New Mexico’s Human Research Protections Office, UNM Health Sciences Center (#16–098). The study was conducted in accordance with the local legislation and institutional requirements. Participants provided direct consent to participate after they reviewed an information sheet.

### Establish validity and shortening objectives

The objective of this project was to shorten the measurement portion of the E^2^ CES from 93 items to approximately 25–30 items to provide actionable data to partnerships in a more flexible and practical manner. Further, these efforts can enhance uptake of the CES in other projects due to reduction in participant burden. We utilized the most recently distributed E^2^ CES instrument with 93 scale items (20) organized across eight higher-order constructs within the four domains of the CBPR model (34). For example, an item for collective empowerment is “Our partnership evaluates together what we have done well and how we can improve our collaboration” and an item for synergy is “We work together well as a partnership.” Scales and constructs in the E^2^ CES have demonstrated strong factorial validity, strong convergent validity, and strong internal consistency. The shortened measure preserves these elements (20).

### Use a conceptual model

The development of the E^2^ CES was guided by the CBPR model (35) and is limited in scope relative to the entire model due to the model’s complexity (36). To stay consistent with the original purpose of designing the E^2^ CES, we used the CBPR model to guide the organization of our surveys and focus groups, as well as our psychometric analyses of convergent validity in creating the shortened measure. That is, choices to remove or retain items ensured that the four domains (context, partnering practices, research-intervention actions, and outcomes) were represented in the final survey.

### Preserve content validity and importance

We invited 40 experts from the E^2^ Think Tank members and E^2^ community partners to participate in a Content Expert and Stakeholder Survey (See Supplementary material 1). The experts included academic and community partners who had been associated with the E^2^ project and thus had familiarity with the original survey and conceptual measure. They also had their own community-academic partnership experience that they drew on to evaluate survey items. Following Gideon et al. (37), we asked respondents to categorize the content of CES items and scales as “least important,” “very important: might be good to include,” or “most important: needs to be included.” Additionally, we asked the participants to rate the items as actionable: “least actionable,” “very actionable: include if there is room,” or “most actionable: need to be included.” We allowed respondents to choose to focus on one or more of the domains of the CBPR model. We also included descriptions of these domains and the definition of the content of each scale. The responses to the items were converted to a 10-point scale (1–10) using the proportion of respondents who rated the items as most importance or most actionable and multiplying by 10.

### Preserve psychometric properties

We preserved psychometric properties by applying analytic techniques used in classical test theory. Scale construction in this approach is a traditional quantitative approach used to test the reliability and validity of scales and their items with an assumption that all observed scores include true and error scores (38, 39). Each CES item and scale were evaluated and ranked in four areas: consistency within scale or construct, convergent validity, ceiling effects, and responsiveness to change (for some items). The original survey was used as part of a post-intervention survey, but since only a subset of scales and items were included, it was not possible to measure responsiveness to change for all scales and items.

A composite ranking for each item and scale was calculated as the mean of the non-missing ranks across the four ranked areas. This ranking was converted to a 10-point scale by multiplying the average item ranking by the proportion of respondents ranking the item content as most important. Rankings for consistency within scale or construct, information, and responsiveness to change were based on a single statistic. In contrast, since the CBPR model has a reciprocal feedback structure across multiple domains, ranking convergent validity involved calculating a composite rank across multiple individual statistics measuring convergent validity. To explore reducing the sensitivity of composite rankings to a trivial difference, “coarse” composite rankings were calculated based on the number of times the statistic for an item or scale exceeds statistics for related items within a ranked area by at least a small effect size.

The statistics for each of the ranked areas at the item level as follows:

- Consistency within scale — “item-rest” correlations between each item score and the average score of the remaining items within a scale were calculated. Following Cohen (40), two correlations will be considered to differ by no more than a small amount if *q* < 0.10.
- Convergent validity — calculating correlations between scores of E^2^ CES items and scale scores of E^2^ SEM constructs in adjacent domains of the CBPR model. Correlations for which Cohen’s *q* < 0.10 will be considered to differ by no more than a small amount.
- Ceiling effects — percent of respondents who selected the highest value for an item.
- Responsiveness to change — Responsiveness indicates the ability of a measure to detect change over time commensurate with the change (and amount) that occurred in the construct (41). We used Cohen’s *d_z_* to calculate and rank the extent of pre/post differences in means of item scores.

### Justify the selection of items and scale

The results from the Content Expert and Stakeholder Survey and the statistical information from the psychometric property testing were used to create a 30-point score for each item (10 on actionable, 10 on content importance, and 10 on statistical importance). We invited participants who completed the survey to participate in one of two Zoom focus groups to review the results and make final recommendations for retention. A summary table of the results was provided to the participants; it included overall rankings and initial recommendations for the disposition of each item and scale. Supplementary material 2 includes the summary table and Supplementary material 3 includes the questions for the participants.

### Validate the short-form version

We tested the PHIRE with the original data set collected with the larger survey (20). We tested the internal consistency, factor structure of the measurement model, and the structural model. Specifically, confirmatory factor analysis and structural equation modeling were performed using Stata 15.0 (42). Fit indices included the Tucker-Lewis Index (TLI) and Standardized Root Mean Square Residual (SRMR).

We completed further validity testing with a new sample due to the fact that the focus group participants suggested some wording changes to items. We administered the shortened version to a network of community health councils in New Mexico. The survey supported the New Mexico Department of Health and its Health Promotion unit to assess their own capacities to collaborate with community partners throughout the state. We examined reliability and correlations amongst the constructs using SPSS 29.0. Additionally, psychometric testing was not administered due to the relatively small sample size.

## Results

### Content validity and psychometric data

Of the 40 invitations, 21 CBPR experts (13 academic and 8 community partners) completed the Content Expert and Stakeholder Survey. There were 11 men and 10 women. Each item received between 14 and 18 responses in the rating of content importance and actionability. The results from the survey are presented in Supplementary material 2. This table also includes the results of statistical importance for the psychometric data.

Each item was scored on a 0–10 scale in three areas, leading to a total possible score of 30. Items scoring 15 or higher were recommended for inclusion in the shortened survey and are highlighted in green in Supplementary material 2. Items scoring 14 were highlighted in purple for possible inclusion; items scoring 13 as the only item within a subscale were also highlighted purpose for possible inclusion.

### Focus groups and final survey creation

Of the 21 CBPR/CEnR experts, 15 also participated in one of the two focus groups: 9 in one and 6 in the other. There were 6 community and 9 academic experts: 9 male and 6 female. The focus groups lasted approximately 60 min. The participants were sent the summary table and explanations of the rankings. The first focus group concentrated on the first two domains of the CBPR model: context and partnering processes. The second group examined intervention-research synergy and outcomes, the latter two domains of the CBPR model.

The focus group participants reviewed the items in each of the scales and discussed whether to retain or drop the item or consider alternative wording changes. The groups recommended retaining a few items that scored below 14 in the composite ratings. In that process, the groups recommended creating items that combined the elements of several items into one item. Notes from each focus group were captured and these notes along with the transcripts were reviewed by the research team.

The research team met to review the recommendations of the focus groups alongside the data in Supplementary material 2. Through a series of multiple meetings, the team created the 30-item Partnering for Health Improvement and Research Equity (PHIRE) survey. These included 14 items that were recommended for inclusion (green) and five items that were marginal (purple) for inclusion from the Supplementary material. Three items that were not originally recommended were retained based on the focus group feedback. Eight items were revised and used elements from either the Key Informant Survey (KIS, the survey for principal investigators/director about project characteristics) related to structural governance (*n* = 3) or used a combination or proxy from the original CES survey (*n* = 8). Many of the items were slightly reworded from the original focusing on the collective partnership (e.g., we) versus individual reflection (e.g., “I”).

### Final confirmation

The initial testing of the original scale was to determine if the shortened version would still fit the original data. While there were wording changes and combinations recommended from the focus group participants, we initially fit the best representative items for the original data set. The details of the original data set are presented elsewhere (20). In brief, the study had 210 projects represented with a principal investigator or designate completing a KIS regarding information about the project and partnership. The respondent also nominated up to three community and one academic partner to complete the E^2^ CES. There were 457 total respondents with the following demographics: 246 White, 71 Black or African American, 66 American Indian/ Alaska Native, 45 Hispanic or Latino, 36 Asian, 11 Native Hawaiian or other Pacific Islander; 36 LGBTQ, 48 low socio-economic status, 26 persons with disabilities, 49 immigrants, and 4 refugees; 99 male and 325 female; 185 community partners and 265 academic partners.

Table 1 displays the psychometric properties of the survey organized around the eight high-order constructs. Cronbach’s alphas were generally high with only one high-order construct having an alpha below 0.70 (structural governance; likely because the items came from the CES and KIS as two levels of data). The factor loadings for items to the higher-order constructs were statistically significant although there were four covariances between items included to improve model fit. TLI and SRMR for the subscales for the high-order constructs were 0.98 or above for TLI and 0.02 or below for SRMR (the subscales for structural governance and future outcomes were saturated models so these fit indices were not available).

**Table 1:** Statistical properties of the PHIRE based on original items.

Link to original item	Higher-order construct and item	CFA	SEM^a^
		β (*SE*)	β (*SE*)
**Partnership Capacity** (*α* = 0.87, TLI = 0.98 SRMR = 0.02)			
Part Capacity 3	1. Legitimacy and credibility in the community	0.65 (0.03)	0.69 (0.03)
Part Capacity 4	2. Ability to bring people together for meetings/activities	0.71 (0.03)	0.72 (0.03)
	Covariance of Legitimacy and Ability	0.58 (0.04)	0.58 (0.04)
Bridging 3	3. The academic partners have the knowledge, skills, and confidence to interact effectively with the community partners.	0.80 (0.02)	0.79 (0.03)
Bridging 1	4. The community partners have the knowledge, skills, and confidence to interact effectively with the academic partners	0.74 (0.03)	0.77 (0.03)
Part Capacity 2 & Bridging 3	5a. Diverse members	0.77 (0.03)	0.76 (0.03)
	5b. The academic partners have the knowledge, skills, and confidence to interact effectively with the community partners.		
**Partnering** (α = 0.81, TLI = 0.99, SRMR = 0.02)			
Trust 2	6. I can rely on the people that I work with on this project.	0.60 (0.04)	0.64 (0.04)
Leadership 1	7. [Our leadership] encourages active participation of academic and community partners in decision making.	0.68 (0.03)	0.80 (0.03)
Dialogue 3	8. We listen to each other.	0.69 (0.04)	0.59 (0.04)
Dialogue 5	9. Even when we do not have total agreement, we reach a kind of consensus that we all accept.	0.78 (0.03)	0.67 (0.04)
	Covariance of Listening and Consensus	0.30 (0.07)	0.44 (0.05)
Mission 3	10. There is general agreement with respect to the priorities of our partnership.	0.66 (0.03)	0.62 (0.04)
**Collective Empowerment** (α = 0.81, TLI = 1.00, SRMR = 0.01)			
Influence 1	11. I have influence over decisions that our partnership makes.	0.42 (0.04)	0.46 (0.05)
Reflexivity 2	12. Our partnership evaluates together what we have done well and how we can improve our collaboration.	0.53 (0.04)	0.57 (0.04)
Principles 2 & Reflection 1	13a. This project facilitates equitable partnerships in all phases of the research.	0.77 (0.02)	0.81 (0.02)
	13b. Our partnership has discussions about our role in promoting strategies to address social and health equity.		
	Covariance of Evaluation and Equitable Partnering	0.38 (0.05)	0.30 (0.06)
Principles 1	14. The project builds on resources and strengths in the community.	0.79 (0.02)	0.79 (0.02)
Principles 8–10	15a. This project is responsive to community histories.	0.87 (0.02)	0.83 (0.02)
	15b. This project integrates the words and language of the community.		
	15c. This project connects with the ways things are done in the community.		
**Structural Governance** (α = 0.66)			
KIS-Research Integrity and Governance Practices	16. Who approved participation in this research project on behalf of the community? (individual community members or not community decision vs. local agency, tribal or local government, health board, tribal IRB, community advisory board)	0.40 (0.04)	0.37 (0.05)
KIS-Hiring and Resource Sharing 6	17. Please enter the percentage of financial resources shared with community partners?	0.64 (0.03)	0.66 (0.03)
KIS-Hiring and Resource Sharing 2	18. Who decides how the financial resources are shared? (mostly community, mostly academic, both)?	1.00 (0.00)	1.00 (0.00)
**Community Engagement in Research Actions** (α = 0.90, TLI = 0.99, SRMR = 0.01)			
Comm Engage 1–5	19a. Grant proposal writing	0.84 (0.02)	0.84 (0.02)
	19b. Background research		
	19c. Developing sampling procedures		
	19d. Designing and implementing the intervention		
	19e. Designing data collection instruments		
Comm Engage 7	20. Interpreting study findings	0.86 (0.02)	0.86 (0.02)
Comm Engage 8, 10, 12	21a. Writing reports and journal articles	0.89 (0.02)	0.89 (0.02)
	21b. Informing the community about research progress and findings		
	21c. Sharing findings with other communities		
Comm Engage 11	22. Informing relevant policy makers about findings	0.71 (0.03)	0.72 (0.03)
	Covariance of Disseminating and Informing	0.56 (0.05)	0.55 (0.05)
**Partnership Synergy**			
Synergy 5	23. We work together well as a partnership.	1.00 (0.00)	1.00 (0.00)
**Benefits** (α = 0.74, TLI = 0.99, SRMR = 0.01)			
Principles 3 & Future Outcomes 9	24a. This project helps all partners involved to grow and learn from one another.	0.85 (0.05)	0.84 (0.02)
	24b. Improved academic ability to integrate community perspectives into research design and methods		
Personal 1–3	25a. [Partners experience personal benefits such as] increased use of your expertise or services by others	0.54 (0.05)	0.56 (0.04)
	25b. [Partners experience personal benefits such as] increased ability to acquire additional financial support		
	25c. [Partners experience personal benefits such as] increased ability to seek formal or informal education		
Power 5	26. [Community members] have the capacity or power to promote research that will benefit the community.	0.48 (0.05)	0.46 (0.05)
Agency 1 & 3	27a. [Organizations involved in this partnership have] enhanced reputation	0.62 (0.05)	0.70 (0.03)
	27b. [Organizations involved in this partnership have] increased use of the agency’s expertise or services by others.		
	Covariance of Personal Benefits and Increased Organization Power	0.35 (0.06)	0.33 (0.05)
**Future Outcomes** (α = 0.83)			
Future Outcomes 3	28. Useful findings for the development of practices, programs, or policies	0.89 (0.03)	0.90 (0.02)
Future Outcomes 10	29. Research better linked to community needs	0.78 (0.03)	0.80 (0.02)
Health Outcomes 1	30. Improve community health	0.70 (0.03)	0.72 (0.03)
**Structural Model**			
	Covariance of Knowledge Co-production and Community-Linked Research		0.56 (0.05)
	Partnership Capacity - > Collective Empowerment		0.75 (0.03)
	Collective Empowerment - > Partnering		0.85 (0.03)
	Partnering - > Partnership Synergy		0.52 (0.10)
	Collective Empowerment - > Partnership Synergy		0.33 (0.23)
	Structural Governance - > Community Engagement		0.16 (0.05)
	Collective Empowerment - > Community Engagement		0.65 (0.04)
	Community Engagement - > Benefits		0.41 (0.04)
	Partnership Synergy - > Benefits		0.55 (0.04)
	Benefits - > Future Outcomes		0.88 (0.02)

CFA = Confirmatory Factor Analysis, SEM = Structural Equation Model.

^a^TLI = 0.92, SRMR = 0.07.

Items with letters following same number were averaged together.

The structural model for the high-order constructs is consistent with the CBPR conceptual model (21, 35), further supporting the conceptual fit of the pragmatic measure with the original conceptual model that guided the development of the longer version. Specifically, these results are consistent with the two paths of the conceptual model. In path one, structural governance is positively associated with community participation which is positively associated with benefits and then to future outcomes. In the second path, partnership capacity is positively associated with collective empowerment, which is positively associated with partnering and then to synergy, benefits, and future outcomes.

The second testing including the administration of the survey to 63 members of community health councils in New Mexico: (a) 45 women, 11 men, and 7 not reporting; (b) 16 were 40 or under, 29 41–60, and 16 older than 60; and (c) 37 white, 18 Latinx, 4 American Indian, and 8 others (numbers do not add up to 63 as participants could select more than one ethnicity).

Table 2 presents the PHIRE with final wording changes. The governance items were not included in the survey because they did not apply with the structure of the health councils. Cronbach’s alphas were high for the remaining subscales with all but one at 0.90 or above. Partnering was at 0.73 and we think this is because we had two negatively phrased items in this scale and upon further review recommended that these be phrased in a positive format. Table 3 presents the correlations among the scales. These are in the expected direction and magnitude given the paths identified in Table 1 and the original structural equation model. Thus, these data support the construct validity of the revised wording changes for the PHIRE.

**Table 2:** Tested PHIRE with wording changes.

Scale	Items	Response options
Partnership capacity (*α* = 0.93)	Does your partnership have any of the following capacities to achieve the project aims? Community legitimacy and trust Ability to bring people together for meetings/activities Academic partners with the skills and experience needed to interact effectively with community partners Community partners with the skills and experience needed to interact effectively with academic partners Diverse partners, reflecting the community	Not at all To a small extent To a moderate extent To a great extent To a very great extent
Partnering (*α* = 0.73)	How much do you agree or disagree that in this partnership: We can rely on each other We (lack) have consensus about the priorities of our partnership Our leadership encourages active participation of all partners in decision making When we have conversations, we often (mis)understand each other Even when we do not have total agreement, we reach a kind of consensus that we all accept	Strongly disagree Disagree Neither agree nor disagree Agree Strongly agree
Collective empowerment (*α* = 0.92)	How much do you agree or disagree that in this partnership: We all have influence over decisions that our partnership makes We evaluate together what we have done well and how we can improve our collaboration We partner equitably while promoting social and health equity Our project builds on resources and strengths in the community Our partnership honors community contributions to knowledge	Not at all To a small extent To a moderate extent To a great extent To a very great extent
Governance and resource sharing (not tested)	This section of the PHIRE survey tool asks about any structures your project has in place to ensure ongoing accountability to community needs and fairness in the sharing of project resources. The term community-based oversight body is used in this section of the survey tool to refer to any group, board, or governing body that has some autonomy from the core research partnership and provides continuing, community-based oversight of the research process. Examples may include a community advisory board, the leadership of a community-based organization, or a local or tribal government. There is a community-based oversight body that ensures that this project benefits the community Community and academic partners decide together how to share the project’s financial resources Resources are shared fairly in this project	Not at all To a small extent To a moderate extent To a great extent To a very great extent I do not know
Community engagement in research actions (*α* = 0.90)	How much have community partners been involved in the following research steps? For steps that have not yet happened, how much will community members be involved? Designing and implementing the intervention and/or study Interpreting study findings Disseminating useful findings for community action and benefit Informing relevant policy makers about findings	Not at all involved Slightly involved Moderately involved Very involved Extremely involved
Partnership synergy	We work together effectively as a community/academic partnership.	Strongly disagree Disagree Neither agree nor disagree Agree Strongly agree
Benefits (*α* = 0.93)	How much do you agree or disagree that as a result of this project: Academic partners have come to value community knowledge more and more All partners experience personal benefits such as enhanced reputation or increased use of skills and expertise by others Community members have increased power to promote research that will benefit the community Organizations involved in this partnership have increased power to improve community health	Strongly disagree Disagree Neither agree nor disagree Agree Strongly agree
Future outcomes (*α* = 0.92)	How much will this project produce: Useful findings for the development of practices, programs, or policies Research better linked to community needs Improved community health	Not at all To a small extent To a moderate extent To a great extent To a very great extent

**Table 3:** Correlation matrix.

Scale	Capacity	Empowerment	Partnering	Comm engagement	Synergy	Benefits
Partnership Capacity						
Collective Empowerment	0.70					
Partnering	0.62	0.74				
Community Engagement in Research Actions	0.75	0.74	0.67			
Partnership Synergy	0.64	0.70	0.70	0.66		
Benefits	0.75	0.68	0.65	0.70	0.73	
Future Outcomes	0.50	0.54	0.38	0.53	0.24	0.71

All relationships significant at 0.01 level except for synergy and future outcomes (0.086).

## Discussion

This study aimed to develop a pragmatic tool for measuring community-academic partnerships in CBPR/CEnR projects related to the CBPR conceptual model that reflects best practices contributing to outcomes. A rigorous six-stage process produced a 30-item instrument across eight higher-order constructs that has strong psychometric properties and construct validity with the original 93-item instrument. This section discusses this measure in the context of the literature of CBPR/CEnR processes and describes the benefits of this novel instrument. We also discuss additional developments in the use of the PHIRE and implications for future research and practices.

CEnR, CBPR, and PEnR have increased in usage and acceptance and as a result, so has the need for instruments to assess and evaluate these approaches. Systematic reviews of instruments (11, 12) have indicated relatively few comprehensive instruments that measure a range of domains in CBPR/CEnR processes and/or that have established psychometric properties. The E^2^ CES is one such tool. In addition, it is only one of three tools that cover all the domains identified by the NAM model (30). The current PHIRE study has further solidified these key domains alongside the original E^2^ CES measures and demonstrates good construct validity and reliability.

Longer instruments, even when they have strong psychometrics, are limited due to practical reasons in many settings. Thus, pragmatic instruments that provide short and effective measurement for practitioners are needed (32). This study established evidence for the PHIRE to support the key criteria for a pragmatic instrument. First, community and academic partners were involved in adapting the instrument to ensure there was support. Second, the shorter version has a relatively low implementation burden for partnerships to assess their CBPR/CEnR contexts, partnering practices, and outcomes in annual evaluations or collective reflection retreats. Third, based on Freirian praxis of cycles of reflection and action, the instrument provides information that can be used by partner teams to assess their strengths and achievements, as well as challenges to make actionable changes in their partnering practices to contribute to desired outcomes (14). Finally, the instrument can be adapted and added to work within a particular community and context. CEnR and CBPR researchers have long recognized the importance of being able to adapt research processes and instruments to fit local cultural contexts (43).

The PHIRE instrument, thus, demonstrates key characteristics from the diffusion of innovation perspective (44). Its primary relative advantage is its brevity—this feature makes it attractive to partnerships that do not want to be burdened with a measurement instrument yet want to have a tool that is valid and reliable. Similarly, the instrument is not complex and is relatively easy to use. A companion self-administered webapp will also soon be available to support partnerships in using the instrument as a tool to strengthen their practice (See text footnote 1). With this paper, the instrument is trialable as it can be downloaded and applied in a pilot setting or within early partnership meetings. Finally, the instrument has flexibility to allow it to be compatible within a specific context. We encourage partnerships to add additional scales or delete ones that are not important to their context and project. Some may want to add full scales in areas of special interest, such as trust, from the CES or another validated instrument to the higher-order shorter PHIRE instrument to provide more depth. As an instrument, however, demonstrating strong innovative properties, the implication is that the PHIRE is a useful and adaptable tool to enhance the practice of CBPR/CEnR.

We are aware of several adaptations to illustrate this benefit. First, engaging partners from the USA and Latin America, our team has developed a Spanish translation of both the CES (Encuesta Comunitaria) and the PHIRE instrument, Fortaleciendo y Uniendo EsfueRzos Transdisciplinarios para Equidad de Salud (FUERTES), for use among Spanish-speaking partnerships (19). In so doing, there were recommendations made to simplify, and change to positive, a few of the items in the English version, also represented in our revised items. They also included a set of items to assess use of community advisory boards. Second, our project team worked in partnership with a couple of Navajo communities in a pilot study to explore “their perspectives about community-engaged research and community well-being from a Diné lens” (45, p.1). The research partners administered the CES but soon learned the need to translate the CES with an interest in the development of a shortened instrument resulting in the PHIRE (46). Third, the long-term NIH-funded Family Listening Program of UNM’s Center for Participatory Research is testing the PHIRE longitudinally with a new partnership of three long-term tribal research teams and three new tribal community advisory boards (47). Finally, other opportunities are in process. Among them is the adaptation of PHIRE to the new NIH ComPASS awardees to assess their partnerships in the design and implementation of structural interventions over time. Further, there is an emerging integration of PHIRE with Community Campus Partnerships for Health assessment of their partnership principles. Future research can test these adaptations and translations as well as explore the further use of the PHIRE in research and practice. It is important to note that while two of these applications included Navajo communities in piloting the PHIRE, the diversity across tribal nations lends itself to CBPR/CEnR under very different contexts and conditions and likely warrants further adaptation across local tribal and urban Native contexts. This caveat is important for other cultural communities as well.

There are several limitations for our study. First, our shortening process is primarily based on the original data set that was used to create the current version of the CES (20). The second data collection does help to alleviate some of this concern. Second, we had some challenges with model convergence that limit some of the tests that can be run. Nonetheless, we were able to provide a robust test to support the validity of the PHIRE. Finally, our community and academic testing was based on members of our own Think Tank who may have some bias related to supporting the CBPR conceptual model. However, these individuals have vast experience in CBPR/CEnR and have developed their own instruments, so this bias is somewhat mitigated by these factors.

## Conclusion

In conclusion, the aim of this study was to develop and test a pragmatic measure for assessing CEnR, CBPR, and PEnR research projects. This study demonstrates that a shorter version of the E^2^ CES is a reliable instrument with construct validity compared to the larger version from which it was derived. The science of CBPR/CEnR and related research is dependent on the development of instruments with strong psychometric properties. However, the practice of CBPR/CEnR is dependent on pragmatic measures that are not a burden to administer and that provide benefit to partnerships interested in strengthening their partnering practices to more effectively research their outcomes. The PHIRE is a straightforward and easy-to-use tool for a range of CBPR/CEnR projects, both research and practical applications. It has a sound conceptual and research foundation, and it is also something that can be adapted and added to fit local cultural contexts.

## Data Availability

The raw data supporting the conclusions of this article will be made available by the authors, without undue reservation.

## Footnotes

^1^ https://engageforequity.org
