# Supplementary material for: A short pragmatic tool for evaluating community engagement: Partnering for Health Improvement and Research Equity
Source: Front Public Health. 2025 Jun 11;13:1539864. doi: 10.3389/fpubh.2025.1539864 (PMC12198686; doi:10.3389/fpubh.2025.1539864)
Supplement: Supplementary file 1 [file Data_Sheet_1.PDF]

CBPR Content Expert and Stakeholder Survey

Instructions

Thank you for taking this survey! This survey is part of the "Shorter is Better: Measuring Community Engaged Research" research study which seeks to: 1) preserve Community Engagement Survey (CES) content validity by obtaining expert input and 2) understand the actionability of CES items and scales.

The terms "content validity" and "actionability" are used in this survey. Content validity of items within a scale refers to the extent to which the given items measure important aspects of the underlying construct or concept under consideration. Actionability refers to the ability of partnerships to take action based on their results from survey items.

This survey also refers to the 4 domains of the CBPR model: Context, Partnership Processes, Intervention & Research, and Outcomes.

Context provides grounding for collaboration on the priority health issue(s). Contextual factors include socio-structural and historical conditions, national and local policies, community and academic capacities, and levels of mutual trust. Partnership Processes are the promising practices that promote equitable contributions from all stakeholders. The science of Intervention & Research processes is influenced by partnering processes and contributes to short-term outputs. Outcomes include both intermediate system, policy, and capacity changes and longer-term changes to community conditions and health outcomes.

There is a "Save & Return Later" button at the bottom of each page of this survey. Clicking this button will allow you access to a survey link that you can use to change or add to your answers. Clicking the "Submit" button at the end of the survey will also allow you access to a survey link that you can use to change or add to your answers. Moreover, once you submit your completed survey, you will receive an email invitation to be randomly selected to participate in a CBPR Content Expert and Stakeholder Focus Group along with a .pdf of your survey responses.

Any information you share with us will be kept private, and will not be shared in any way that could identify you or your responses to this survey. If you have any questions or comments about this survey, please let us know by email to CPR@salud.unm.edu or by calling Blake Boursaw at 505-272-4465.

Thank you for your time and attention!

This survey asks questions about items and constructs across all 4 domains of the CBPR model. Which domains of the CBPR model do you want to focus on? Please check all that apply.	☐ Context (2 sections, 8 items)<br>☐ Partnership Processes (10 sections, 41 items)<br>☐ Intervention & Research (2 sections, 18 items)<br>☐ Outcomes (6 sections, 26 items)
This survey includes questions with response options that emphasize actionability and with response options that emphasize content validity. Which types of response options do you want to see? Please check all that apply.	☐ Actionability<br>☐ Content validity

Title: "Community Context and Capacity"Response options: Response options for Community Context and Capacity items range from "not at all" to "to a complete extent".Domain: Section 1 of 2 of ContextDescription: Community Context and Capacity items measure the extent to which "the partnership has the ability to build from community capacities and histories of advocacy to confront inequitable community conditions".Instructions: Choose one importance to stakeholders and one content validity response option for each item. Recall that, across this survey, you should choose about 1/3 of items to be in each importance to stakeholders and each content validity response category.

	Least actionable	Very actionable: include if there is room	Most actionable: needs to be included	Least important to content	Very important to content: include if there is room	Most important to content: needs to be included
Item 1. The community or communities participating in this project have a history of organizing services or events.	☐	☐	☐	☐	☐	☐
Item 2. The community or communities participating in this project have a history of advocating for social or health equity.	☐	☐	☐	☐	☐	☐
Item 3. By working together, people in the community or communities participating in this project have previously influenced decisions that affected their communities.	☐	☐	☐	☐	☐	☐

Overall, how important is it that at least one "Community Context and Capacity" item remains in a shortened version of the Community Engagement Survey?

○ Least important
○ Very important
○ Most important

Title: "Partnership Capacity"Response options: Response options for Partnership Capacity items range from "not at all" to "to a complete extent".Domain: Section 2 of 2 of ContextDescription: Partnership Capacity items measure the extent to which the partnership has "foundational resources and skills necessary for the partnership to achieve project goals".Instructions: Choose one importance to stakeholders and one content validity response option for each item. Recall that, across this survey, you should choose about 1/3 of items to be in each importance to stakeholders and each content validity response category.

	Least actionable	Very actionable: include if there is room	Most actionable: needs to be included	Least important to content	Very important to content: include if there is room	Most important to content: needs to be included
Item 1. Skills and expertise	☐	☐	☐	☐	☐	☐
Item 2. Diverse members	☐	☐	☐	☐	☐	☐
Item 3. Legitimacy and credibility in the community	☐	☐	☐	☐	☐	☐
Item 4. Ability to bring people together for meetings/activities	☐	☐	☐	☐	☐	☐
Item 5. Connections to relevant stakeholders	☐	☐	☐	☐	☐	☐

Overall, how important is it that at least one "Partnership Capacity" item remains in a shortened version of the Community Engagement Survey?

◯ Least important
◯ Very important
◯ Most important

Title: "Bridging Differences"Response options: Response options for Bridging Differences items range from "not at all" to "to a complete extent".Domain: Section 1 of 10 of Partnership ProcessesDescription: Bridging Differences items measure the extent to which the partnership has "the capacity to work across difference and also includes academic team members sharing similar cultural, racial-ethnic, identity backgrounds to community partners".Instructions: Choose one importance to stakeholders and one content validity response option for each item. Recall that, across this survey, you should choose about 1/3 of items to be in each importance to stakeholders and each content validity response category.

	Least actionable	Very actionable: include if there is room	Most actionable: needs to be included	Least important to content	Very important to content: include if there is room	Most important to content: needs to be included
Item 1. The community partners (such as patients, community members, or organizations) have the knowledge, skills, and confidence to interact effectively with the academic partners (such as individuals from universities).	☐	☐	☐	☐	☐	☐
Item 2. The academic partners have members who are from a similar background as the community partners.	☐	☐	☐	☐	☐	☐
Item 3. The academic partners have the knowledge, skills, and confidence to interact effectively with the community partners.	☐	☐	☐	☐	☐	☐

Overall, how important is it that at least one "Bridging Differences" item remains in a shortened version of the Community Engagement Survey?

- ○ Least important
- ○ Very important
- ○ Most important

Title: "Mission and Strategies"Response options: Response options for Mission and Strategies items range from "completely disagree" to "completely agree".Domain: Section 2 of 10 of Partnership ProcessesDescription: Mission and Strategies items measure the extent to which the partnership has "shared values and understandings of problems, mission, priorities, and strategies".Instructions: Choose one importance to stakeholders and one content validity response option for each item. Recall that, across this survey, you should choose about 1/3 of items to be in each importance to stakeholders and each content validity response category.

	Least actionable	Very actionable: include if there is room	Most actionable: needs to be included	Least important to content	Very important to content: include if there is room	Most important to content: needs to be included
Item 1. Members of our partnership have a clear and shared understanding of the problems we are trying to address.	☐	☐	☐	☐	☐	☐
Item 2. Members can generally state the mission and goals of our partnership.	☐	☐	☐	☐	☐	☐
Item 3. There is general agreement with respect to the priorities of our partnership.	☐	☐	☐	☐	☐	☐
Item 4. There is general agreement on the strategies our partnership should use in pursuing its priorities.	☐	☐	☐	☐	☐	☐

Overall, how important is it that at least one "Mission and Strategies" item remains in a shortened version of the Community Engagement Survey?

○ Least important
○ Very important
○ Most important

Title: "Influence in the Partnership"Response options: Response options for Influence in the Partnership items range from "completely disagree" to "completely agree".Domain: Section 3 of 10 of Partnership ProcessesDescription: Influence in the Partnership items measure "the perception of how individual team members feel about their ability to contribute to decisions in the research team context".Instructions: Choose one importance to stakeholders and one content validity response option for each item. Recall that, across this survey, you should choose about 1/3 of items to be in each importance to stakeholders and each content validity response category.

	Least actionable	Very actionable: include if there is room	Most actionable: needs to be included	Least important to content	Very important to content: include if there is room	Most important to content: needs to be included
Item 1. I have influence over decisions that this partnership makes.	☐	☐	☐	☐	☐	☐
Item 2. My involvement influences the partnership to be more responsive to the community.	☐	☐	☐	☐	☐	☐
Item 3. I am able to influence the work on this project.	☐	☐	☐	☐	☐	☐

Overall, how important is it that at least one "Influence in the Partnership" item remains in a shortened version of the Community Engagement Survey?

◯ Least important
◯ Very important
◯ Most important

Title: "Participatory Decision Making"Response options: Response options for Participatory Decision Making items range from "completely disagree" to "completely agree".Domain: Section 4 of 10 of Partnership ProcessesDescription: Participatory Decision Making items measure the extent to which the partnership has "decision making that takes all opinions into account, though there are multiple ways to achieve high levels of participation".Instructions: Choose one importance to stakeholders and one content validity response option for each item. Recall that, across this survey, you should choose about 1/3 of items to be in each importance to stakeholders and each content validity response category.

	Least actionable	Very actionable: include if there is room	Most actionable: needs to be included	Least important to content	Very important to content: include if there is room	Most important to content: needs to be included
Item 1. I feel comfortable with the way decisions are made in this partnership.	☐	☐	☐	☐	☐	☐
Item 2. When decisions are made, I support the decisions made by other partners in this partnership.	☐	☐	☐	☐	☐	☐
Item 3. When decisions are made, I feel that my opinion is taken into consideration by other partners in this partnership.	☐	☐	☐	☐	☐	☐

Overall, how important is it that at least one "Participatory Decision Making" item remains in a shortened version of the Community Engagement Survey?

◯ Least important
◯ Very important
◯ Most important

Title: "Quality of Dialogue. How much do you agree or disagree that this partnership has conversations where:"Response options: Response options for Quality of Dialogue items range from "completely disagree" to "completely agree".Domain: Section 5 of 10 of Partnership ProcessesDescription: Quality of Dialogue items measure the extent to which "all partners listen and participate in dialogue with each other so that all opinions and knowledge are valued, and community members feel their voices are equally valued" and "community and academic partners interact, negotiate, and manage conflicts, tensions, and frictions that emerge in the partnered research".Instructions: Choose one importance to stakeholders and one content validity response option for each item. Recall that, across this survey, you should choose about 1/3 of items to be in each importance to stakeholders and each content validity response category.

	Least actionable	Very actionable: include if there is room	Most actionable: needs to be included	Least important to content	Very important to content: include if there is room	Most important to content: needs to be included
Item 1. We show positive attitudes towards one another.	☐	☐	☐	☐	☐	☐
Item 2. Everyone in our partnership participates in our meetings.	☐	☐	☐	☐	☐	☐
Item 3. We listen to each other.	☐	☐	☐	☐	☐	☐
Item 4. When conflicts occur, we work together to resolve them.	☐	☐	☐	☐	☐	☐
Item 5. Even when we don't have total agreement, we reach a kind of consensus that we all accept.	☐	☐	☐	☐	☐	☐

Overall, how important is it that at least one "Quality of Dialogue" item remains in a shortened version of the Community Engagement Survey?

◯ Least important
◯ Very important
◯ Most important

Title: "Reflexivity"Response options: Response options for Reflexivity items range from "completely disagree" to "completely agree".Domain: Section 6 of 10 of Partnership ProcessesDescription: Reflexivity items measure the extent to which the partnership has the "capacity to evaluate and reflect on their own partnership processes in order to seek continual improvement; and to recognize the challenges of addressing issues of equity, power, and privilege in their research processes".Instructions: Choose one importance to stakeholders and one content validity response option for each item. Recall that, across this survey, you should choose about 1/3 of items to be in each importance to stakeholders and each content validity response category.

	Least actionable	Very actionable: include if there is room	Most actionable: needs to be included	Least important to content	Very important to content: include if there is room	Most important to content: needs to be included
Item 1. Our partnership has discussions about our role in promoting strategies to address social and health equity.	☐	☐	☐	☐	☐	☐
Item 2. Our partnership evaluates together what we've done well and how we can improve our collaboration.	☐	☐	☐	☐	☐	☐
Item 3. Our partnership reflects on issues of power and privilege within our partnership.	☐	☐	☐	☐	☐	☐

Overall, how important is it that at least one "Reflexivity" item remains in a shortened version of the Community Engagement Survey?

◯ Least important
◯ Very important
◯ Most important

Title: "Leadership. How well does the leadership for the partnership:"Response options: Response options for Leadership items range from "not at all well" to "completely well".Domain: Section 7 of 10 of Partnership ProcessesDescription: Leadership items measure the extent to which the leadership for the partnership "honors knowledge and encourages participation from all partners, and supports development of community leaders as equal partners".Instructions: Choose one importance to stakeholders and one content validity response option for each item. Recall that, across this survey, you should choose about 1/3 of items to be in each importance to stakeholders and each content validity response category.

	Least actionable	Very actionable: include if there is room	Most actionable: needs to be included	Least important to content	Very important to content: include if there is room	Most important to content: needs to be included
Item 1. Encourage active participation of academic and community partners in decision making	☐	☐	☐	☐	☐	☐
Item 2. Communicate the goals of the project	☐	☐	☐	☐	☐	☐
Item 3. Foster respect between partners	☐	☐	☐	☐	☐	☐
Item 4. Help the partners be creative and look at things differently	☐	☐	☐	☐	☐	☐

Overall, how important is it that at least one "Leadership" item remains in a shortened version of the Community Engagement Survey?

◯ Least important
◯ Very important
◯ Most important

Title: "Resource Use. How well does your project use:"Response options: Response options for Resource Use items range from "not at all well" to "completely well".Domain: Section 8 of 10 of Partnership ProcessesDescription: Resource Use items "reflect partners? perceptions of how effective the project is at using the partnership?s resources and time".Instructions: Choose one importance to stakeholders and one content validity response option for each item. Recall that, across this survey, you should choose about 1/3 of items to be in each importance to stakeholders and each content validity response category.

	Least actionable	Very actionable: include if there is room	Most actionable: needs to be included	Least important to content	Very important to content: include if there is room	Most important to content: needs to be included
Item 1. The partnership's financial resources	☐	☐	☐	☐	☐	☐
Item 2. The partnership's in-kind resources	☐	☐	☐	☐	☐	☐
Item 3. The partners' time	☐	☐	☐	☐	☐	☐

Overall, how important is it that at least one "Resource Use" item remains in a shortened version of the Community Engagement Survey?

◯ Least important
◯ Very important
◯ Most important

Title: "Trust. How much do you agree or disagree with these statements about the level of trust between partnership members?"Response options: Response options for Trust items range from "completely disagree" to "completely agree".Domain: Section 9 of 10 of Partnership ProcessesDescription: Trust items measure the partnership's "trust development" which is "dynamic" and "rests on participation (showing up), effective communication, and commitment to common goals".Instructions: Choose one importance to stakeholders and one content validity response option for each item. Recall that, across this survey, you should choose about 1/3 of items to be in each importance to stakeholders and each content validity response category.

	Least actionable	Very actionable: include if there is room	Most actionable: needs to be included	Least important to content	Very important to content: include if there is room	Most important to content: needs to be included
Item 1. I trust the decisions others make about issues that are important to our projects.	☐	☐	☐	☐	☐	☐
Item 2. I can rely on the people that I work with on this project.	☐	☐	☐	☐	☐	☐
Item 3. People in this partnership have a lot of confidence in one another.	☐	☐	☐	☐	☐	☐

Overall, how important is it that at least one "Trust" item remains in a shortened version of the Community Engagement Survey?

◯ Least important
◯ Very important
◯ Most important

Title: "Community Engagement Principles. Does this project reflect the following Community Based Participatory Research (CBPR) principles?"Response options: Response options for Community Engagement Principles items range from "not at all" to "to a complete extent".Domain: Section 10 of 10 of Partnership ProcessesDescription: Community Engagement Principles items measure "the degree to which academic and community partners agree with principles of engagement in terms of commitment to partners, partnership, and community well-being" and "reflect how individual team members feel the research project integrates community culture(s), history and understandings in the research and intervention design and implementation".Instructions: Choose one importance to stakeholders and one content validity response option for each item. Recall that, across this survey, you should choose about 1/3 of items to be in each importance to stakeholders and each content validity response category.

	Least actionable	Very actionable: include if there is room	Most actionable: needs to be included	Least important to content	Very important to content: include if there is room	Most important to content: needs to be included
Item 1. This project builds on resources and strengths in the community.	☐	☐	☐	☐	☐	☐
Item 2. This project facilitates equitable partnerships in all phases of the research.	☐	☐	☐	☐	☐	☐
Item 3. This project helps all partners involved to grow and learn from one another.	☐	☐	☐	☐	☐	☐
Item 4. This project balances research and social action for the mutual benefit of all partners.	☐	☐	☐	☐	☐	☐
Item 5. This project emphasizes the factors that are important to the community (e.g., environmental and social factors) which affect well-being.	☐	☐	☐	☐	☐	☐
Item 6. This project communicates knowledge and findings to all partners and involves all partners in the dissemination process.	☐	☐	☐	☐	☐	☐
Item 7. This project views CBPR or community engaged research as a long term process and a long term commitment.	☐	☐	☐	☐	☐	☐

Item 8. This project is responsive to community histories.	☐	☐	☐	☐	☐	☐
Item 9. This project integrates the words and language of the community.	☐	☐	☐	☐	☐	☐
Item 10. This project connects with the ways things are done in the community.	☐	☐	☐	☐	☐	☐

Overall, how important is it that at least one "Community Engagement Principles" item remains in a shortened version of the Community Engagement Survey?

- ◯ Least important
- ◯ Very important
- ◯ Most important

Title: "Community Engagement in Research Project. How much have community partners been involved in the following research steps? For steps that have not yet happened, how much will community members be involved?"Response options: Response options for Community Engagement in Research Project items range from "not at all involved" to "completely involved".Domain: Section 1 of 2 of Intervention & ResearchDescription: Community Engagement in Research Project items measure the extent to which "community members participate in all phases of the research".Instructions: Choose one importance to stakeholders and one content validity response option for each item. Recall that, across this survey, you should choose about 1/3 of items to be in each importance to stakeholders and each content validity response category.

	Least actionable	Very actionable: include if there is room	Most actionable: needs to be included	Least important to content	Very important to content: include if there is room	Most important to content: needs to be included
Item 1. Grant proposal writing	☐	☐	☐	☐	☐	☐
Item 2. Background research	☐	☐	☐	☐	☐	☐
Item 3. Developing sampling procedures	☐	☐	☐	☐	☐	☐
Item 4. Designing and implementing the intervention	☐	☐	☐	☐	☐	☐
Item 5. Designing data collection instruments (such as interviews or surveys)	☐	☐	☐	☐	☐	☐
Item 6. Collecting primary data	☐	☐	☐	☐	☐	☐
Item 7. Interpreting study findings	☐	☐	☐	☐	☐	☐
Item 8. Writing reports and journal articles	☐	☐	☐	☐	☐	☐
Item 9. Giving presentations at meetings and conferences	☐	☐	☐	☐	☐	☐
Item 10. Informing the community about research progress and findings	☐	☐	☐	☐	☐	☐
Item 11. Informing relevant policy makers about findings	☐	☐	☐	☐	☐	☐
Item 12. Sharing findings with other communities	☐	☐	☐	☐	☐	☐
Item 13. Producing useful findings for community action and benefit	☐	☐	☐	☐	☐	☐

Overall, how important is it that at least one "Community Engagement in Research Project" item remains in a shortened version of the Community Engagement Survey?

- ◯ Least important
- ◯ Very important
- ◯ Most important

Title: "Partnership Synergy. Do you and your partners:"Response options: Response options for Partnership Synergy items range from "not at all" to "to a complete extent".Domain: Section 2 of 2 of Intervention & ResearchDescription: Partnership Synergy items measure the extent to which the partnership has the "ability to develop shared goals and strategies, recognize challenges and needs, and work together effectively".Instructions: Choose one importance to stakeholders and one content validity response option for each item. Recall that, across this survey, you should choose about 1/3 of items to be in each importance to stakeholders and each content validity response category.

	Least actionable	Very actionable: include if there is room	Most actionable: needs to be included	Least important to content	Very important to content: include if there is room	Most important to content: needs to be included
Item 1. Develop goals that are widely understood and supported in this partnership	☐	☐	☐	☐	☐	☐
Item 2. Develop strategies that are most likely to work for the community or stakeholders as a whole	☐	☐	☐	☐	☐	☐
Item 3. Recognize challenges and come up with good solutions	☐	☐	☐	☐	☐	☐
Item 4. Respond to the needs and problems of your constituency or community as a whole	☐	☐	☐	☐	☐	☐
Item 5. Work together well as a partnership	☐	☐	☐	☐	☐	☐

Overall, how important is it that at least one "Partnership Synergy" item remains in a shortened version of the Community Engagement Survey?

- ◯ Least important
- ◯ Very important
- ◯ Most important

Title: "Agency Outcomes. How much do or will the community or clinical organizations in this partnership enjoy the following benefits?"Response options: Response options for Agency Outcomes items range from "not at all" to "to a complete extent".Domain: Section 1 of 6 of OutcomesDescription: Agency Outcomes items measure the extent to which the partnership has "strengthened skills of community and partner agencies to enhance their reputation, to utilize their expertise, and to affect public policy".Instructions: Choose one importance to stakeholders and one content validity response option for each item. Recall that, across this survey, you should choose about 1/3 of items to be in each importance to stakeholders and each content validity response category.

	Least actionable	Very actionable: include if there is room	Most actionable: needs to be included	Least important to content	Very important to content: include if there is room	Most important to content: needs to be included
Item 1. Enhanced reputation	☐	☐	☐	☐	☐	☐
Item 2. Enhanced ability to affect public policy	☐	☐	☐	☐	☐	☐
Item 3. Increased use of the agency's expertise or services by others	☐	☐	☐	☐	☐	☐

Overall, how important is it that at least one "Agency Outcomes" item remains in a shortened version of the Community Engagement Survey?

◯ Least important
◯ Very important
◯ Most important

Title: "Personal Advantages. Do you or will you enjoy the following benefits from participating in this partnership?"Response options: Response options for Personal Advantages items range from "not at all" to "to a complete extent".Domain: Section 2 of 6 of OutcomesDescription: Personal Advantages items measure the extent to which individual partnership members are "feeling an enhanced sense of expertise and skills and enhanced support for more education".Instructions: Choose one importance to stakeholders and one content validity response option for each item. Recall that, across this survey, you should choose about 1/3 of items to be in each importance to stakeholders and each content validity response category.

	Least actionable	Very actionable: include if there is room	Most actionable: needs to be included	Least important to content	Very important to content: include if there is room	Most important to content: needs to be included
Item 1. Increased use of your expertise or services by others	☐	☐	☐	☐	☐	☐
Item 2. Increased ability to acquire additional financial support	☐	☐	☐	☐	☐	☐
Item 3. Increased ability to seek formal or informal education	☐	☐	☐	☐	☐	☐

Overall, how important is it that at least one "Personal Advantages" item remains in a shortened version of the Community Engagement Survey?

◯ Least important
◯ Very important
◯ Most important

Title: "Power Relations in Research. How much do you agree or disagree that community members:"Response options: Response options for Power Relations in Research items range from "completely disagree" to "completely agree".Domain: Section 3 of 6 of OutcomesDescription: Power Relations in Research items measure the extent to which "community members feel that power is shared equally in the research process".Instructions: Choose one importance to stakeholders and one content validity response option for each item. Recall that, across this survey, you should choose about 1/3 of items to be in each importance to stakeholders and each content validity response category.

	Least actionable	Very actionable: include if there is room	Most actionable: needs to be included	Least important to content	Very important to content: include if there is room	Most important to content: needs to be included
Item 1. Have increased participation in the research process	☐	☐	☐	☐	☐	☐
Item 2. Are able to talk about the project with groups or in other settings, such as community or political meetings	☐	☐	☐	☐	☐	☐
Item 3. Can apply the findings of the research to practices and programs in the community	☐	☐	☐	☐	☐	☐
Item 4. Can voice their opinions about research in front of researchers	☐	☐	☐	☐	☐	☐
Item 5. Have the capacity or power to promote research that will benefit the community	☐	☐	☐	☐	☐	☐

Overall, how important is it that at least one "Power Relations in Research" item remains in a shortened version of the Community Engagement Survey?

◯ Least important
◯ Very important
◯ Most important

Title: "Project Sustainability. How much do you agree or disagree that:"Response options: Response options for Project Sustainability items range from "completely disagree" to "completely agree".Domain: Section 4 of 6 of OutcomesDescription: Project Sustainability items measure the extent to which "partnership members are engaged regardless of funding and the partnership evaluates funding opportunities strategically".Instructions: Choose one importance to stakeholders and one content validity response option for each item. Recall that, across this survey, you should choose about 1/3 of items to be in each importance to stakeholders and each content validity response category.

	Least actionable	Very actionable: include if there is room	Most actionable: needs to be included	Least important to content	Very important to content: include if there is room	Most important to content: needs to be included
Item 1. I am committed to sustaining the community-academic relationship with no or low funding.	☐	☐	☐	☐	☐	☐
Item 2. This project is likely to continue forward after this funding is over.	☐	☐	☐	☐	☐	☐
Item 3. Our partnership carefully evaluates funding opportunities to make sure they meet both community and academic partners' needs.	☐	☐	☐	☐	☐	☐

Overall, how important is it that at least one "Project Sustainability" item remains in a shortened version of the Community Engagement Survey?

◯ Least important
◯ Very important
◯ Most important

Title: "Health Outcomes"Response options: Response options for Health Outcomes items range from "not at all" to "to a complete extent".Domain: Section 5 of 6 of OutcomesDescription: Health Outcomes items measure the strength of "a partnership?s assessment that its efforts will lead to improved health in the community, along with improved health behaviors of community members".Instructions: Choose one importance to stakeholders and one content validity response option for each item. Recall that, across this survey, you should choose about 1/3 of items to be in each importance to stakeholders and each content validity response category.

	Least actionable	Very actionable: include if there is room	Most actionable: needs to be included	Least important to content	Very important to content: include if there is room	Most important to content: needs to be included
Item 1. How much do you think this project will improve the health of the community?	☐	☐	☐	☐	☐	☐
Item 2. How much do you think this project will improve the health behaviors of community members?	☐	☐	☐	☐	☐	☐

Overall, how important is it that at least one "Health Outcomes" item remains in a shortened version of the Community Engagement Survey?

◯ Least important
◯ Very important
◯ Most important

Title: "Future Community-Level, Research, and Policy Outcomes. How much WILL this project produce:"Response options: Response options for Future Community-Level, Research, and Policy Outcomes items range from "not at all" to "to a complete extent".Domain: Section 6 of 6 of OutcomesDescription: Future Community-Level, Research, and Policy Outcomes items measure the partnership's confidence in their ability to "reinforce cultural identity or pride, experience broad social impacts, and produce a better overall community environment", "link research efforts to community needs with an improved ability of academic partners to integrate community perspectives into research design and methods", and have efforts that "lead to policy changes".Instructions: Choose one importance to stakeholders and one content validity response option for each item. Recall that, across this survey, you should choose about 1/3 of items to be in each importance to stakeholders and each content validity response category.

	Least actionable	Very actionable: include if there is room	Most actionable: needs to be included	Least important to content	Very important to content: include if there is room	Most important to content: needs to be included
Item 1. Better coordination between agencies, researchers, and community groups	☐	☐	☐	☐	☐	☐
Item 2. Changes in the nature of debates about important health issues in the community	☐	☐	☐	☐	☐	☐
Item 3. Useful findings for the development of community practices, programs, or policies	☐	☐	☐	☐	☐	☐
Item 4. Changes in policy	☐	☐	☐	☐	☐	☐
Item 5. Changes in clinical practices	☐	☐	☐	☐	☐	☐
Item 6. Better overall environment in the community	☐	☐	☐	☐	☐	☐
Item 7. Reinforced cultural identity or pride	☐	☐	☐	☐	☐	☐
Item 8. Broad social impacts	☐	☐	☐	☐	☐	☐
Item 9. Improved academic ability to integrate community perspectives into research design and methods	☐	☐	☐	☐	☐	☐
Item 10. Research better linked to community needs	☐	☐	☐	☐	☐	☐

Overall, how important is it that at least one "Future Community-Level, Research, and Policy Outcomes" item remains in a shortened version of the Community Engagement Survey?

○ Least important
○ Very important
○ Most important

Title: "Community Context and Capacity"Response options: Response options for Community Context and Capacity items range from "not at all" to "to a complete extent".Domain: Section 1 of 2 of ContextDescription: Community Context and Capacity items measure the extent to which "the partnership has the ability to build from community capacities and histories of advocacy to confront inequitable community conditions".Instructions: Choose one importance to stakeholders response option for each item. Recall that, across this survey, you should choose about 1/3 of items to be in each importance to stakeholders response category.

	Least actionable	Very actionable: include if there is room	Most actionable: needs to be included
Item 1. The community or communities participating in this project have a history of organizing services or events.	◯	◯	◯
Item 2. The community or communities participating in this project have a history of advocating for social or health equity.	◯	◯	◯
Item 3. By working together, people in the community or communities participating in this project have previously influenced decisions that affected their communities.	◯	◯	◯

Overall, how important is it that at least one "Community Context and Capacity" item remains in a shortened version of the Community Engagement Survey?

◯ Least important
◯ Very important
◯ Most important

Title: "Partnership Capacity"Response options: Response options for Partnership Capacity items range from "not at all" to "to a complete extent".Domain: Section 2 of 2 of ContextDescription: Partnership Capacity items measure the extent to which the partnership has "foundational resources and skills necessary for the partnership to achieve project goals".Instructions: Choose one importance to stakeholders response option for each item. Recall that, across this survey, you should choose about 1/3 of items to be in each importance to stakeholders response category.

	Least actionable	Very actionable: include if there is room	Most actionable: needs to be included
Item 1. Skills and expertise	○	○	○
Item 2. Diverse members	○	○	○
Item 3. Legitimacy and credibility in the community	○	○	○
Item 4. Ability to bring people together for meetings/activities	○	○	○
Item 5. Connections to relevant stakeholders	○	○	○

Overall, how important is it that at least one "Partnership Capacity" item remains in a shortened version of the Community Engagement Survey?

○ Least important
○ Very important
○ Most important

Title: "Bridging Differences"Response options: Response options for Bridging Differences items range from "not at all" to "to a complete extent".Domain: Section 1 of 10 of Partnership ProcessesDescription: Bridging Differences items measure the extent to which the partnership has "the capacity to work across difference and also includes academic team members sharing similar cultural, racial-ethnic, identity backgrounds to community partners".Instructions: Choose one importance to stakeholders response option for each item. Recall that, across this survey, you should choose about 1/3 of items to be in each importance to stakeholders response category.

	Least actionable	Very actionable: include if there is room	Most actionable: needs to be included
Item 1. The community partners (such as patients, community members, or organizations) have the knowledge, skills, and confidence to interact effectively with the academic partners (such as individuals from universities).	○	○	○
Item 2. The academic partners have members who are from a similar background as the community partners.	○	○	○
Item 3. The academic partners have the knowledge, skills, and confidence to interact effectively with the community partners.	○	○	○

Overall, how important is it that at least one "Bridging Differences" item remains in a shortened version of the Community Engagement Survey?

○ Least important
○ Very important
○ Most important

Title: "Mission and Strategies"Response options: Response options for Mission and Strategies items range from "completely disagree" to "completely agree".Domain: Section 2 of 10 of Partnership ProcessesDescription: Mission and Strategies items measure the extent to which the partnership has "shared values and understandings of problems, mission, priorities, and strategies".Instructions: Choose one importance to stakeholders response option for each item. Recall that, across this survey, you should choose about 1/3 of items to be in each importance to stakeholders response category.

	Least actionable	Very actionable: include if there is room	Most actionable: needs to be included
Item 1. Members of our partnership have a clear and shared understanding of the problems we are trying to address.	○	○	○
Item 2. Members can generally state the mission and goals of our partnership.	○	○	○
Item 3. There is general agreement with respect to the priorities of our partnership.	○	○	○
Item 4. There is general agreement on the strategies our partnership should use in pursuing its priorities.	○	○	○

Overall, how important is it that at least one "Mission and Strategies" item remains in a shortened version of the Community Engagement Survey?

○ Least important
○ Very important
○ Most important

Title: "Influence in the Partnership"Response options: Response options for Influence in the Partnership items range from "completely disagree" to "completely agree".Domain: Section 3 of 10 of Partnership ProcessesDescription: Influence in the Partnership items measure "the perception of how individual team members feel about their ability to contribute to decisions in the research team context".Instructions: Choose one importance to stakeholders response option for each item. Recall that, across this survey, you should choose about 1/3 of items to be in each importance to stakeholders response category.

	Least actionable	Very actionable: include if there is room	Most actionable: needs to be included
Item 1. I have influence over decisions that this partnership makes.	○	○	○
Item 2. My involvement influences the partnership to be more responsive to the community.	○	○	○
Item 3. I am able to influence the work on this project.	○	○	○

Overall, how important is it that at least one "Influence in the Partnership" item remains in a shortened version of the Community Engagement Survey?

○ Least important
○ Very important
○ Most important

Title: "Participatory Decision Making"Response options: Response options for Participatory Decision Making items range from "completely disagree" to "completely agree".Domain: Section 4 of 10 of Partnership ProcessesDescription: Participatory Decision Making items measure the extent to which the partnership has "decision making that takes all opinions into account, though there are multiple ways to achieve high levels of participation".Instructions: Choose one importance to stakeholders response option for each item. Recall that, across this survey, you should choose about 1/3 of items to be in each importance to stakeholders response category.

	Least actionable	Very actionable: include if there is room	Most actionable: needs to be included
Item 1. I feel comfortable with the way decisions are made in this partnership.	◯	◯	◯
Item 2. When decisions are made, I support the decisions made by other partners in this partnership.	◯	◯	◯
Item 3. When decisions are made, I feel that my opinion is taken into consideration by other partners in this partnership.	◯	◯	◯

Overall, how important is it that at least one "Participatory Decision Making" item remains in a shortened version of the Community Engagement Survey?

◯ Least important
◯ Very important
◯ Most important

Title: "Quality of Dialogue. How much do you agree or disagree that this partnership has conversations where:"Response options: Response options for Quality of Dialogue items range from "completely disagree" to "completely agree".Domain: Section 5 of 10 of Partnership ProcessesDescription: Quality of Dialogue items measure the extent to which "all partners listen and participate in dialogue with each other so that all opinions and knowledge are valued, and community members feel their voices are equally valued" and "community and academic partners interact, negotiate, and manage conflicts, tensions, and frictions that emerge in the partnered research".Instructions: Choose one importance to stakeholders response option for each item. Recall that, across this survey, you should choose about 1/3 of items to be in each importance to stakeholders response category.

	Least actionable	Very actionable: include if there is room	Most actionable: needs to be included
Item 1. We show positive attitudes towards one another.	○	○	○
Item 2. Everyone in our partnership participates in our meetings.	○	○	○
Item 3. We listen to each other.	○	○	○
Item 4. When conflicts occur, we work together to resolve them.	○	○	○
Item 5. Even when we don't have total agreement, we reach a kind of consensus that we all accept.	○	○	○

Overall, how important is it that at least one "Quality of Dialogue" item remains in a shortened version of the Community Engagement Survey?

○ Least important
○ Very important
○ Most important

Title: "Reflexivity"Response options: Response options for Reflexivity items range from "completely disagree" to "completely agree".Domain: Section 6 of 10 of Partnership ProcessesDescription: Reflexivity items measure the extent to which the partnership has the "capacity to evaluate and reflect on their own partnership processes in order to seek continual improvement; and to recognize the challenges of addressing issues of equity, power, and privilege in their research processes".Instructions: Choose one importance to stakeholders response option for each item. Recall that, across this survey, you should choose about 1/3 of items to be in each importance to stakeholders response category.

	Least actionable	Very actionable: include if there is room	Most actionable: needs to be included
Item 1. Our partnership has discussions about our role in promoting strategies to address social and health equity.	○	○	○
Item 2. Our partnership evaluates together what we've done well and how we can improve our collaboration.	○	○	○
Item 3. Our partnership reflects on issues of power and privilege within our partnership.	○	○	○

Overall, how important is it that at least one "Reflexivity" item remains in a shortened version of the Community Engagement Survey?

○ Least important
○ Very important
○ Most important

Title: "Leadership. How well does the leadership for the partnership:"Response options: Response options for Leadership items range from "not at all well" to "completely well".Domain: Section 7 of 10 of Partnership ProcessesDescription: Leadership items measure the extent to which the leadership for the partnership "honors knowledge and encourages participation from all partners, and supports development of community leaders as equal partners".Instructions: Choose one importance to stakeholders response option for each item. Recall that, across this survey, you should choose about 1/3 of items to be in each importance to stakeholders response category.

	Least actionable	Very actionable: include if there is room	Most actionable: needs to be included
Item 1. Encourage active participation of academic and community partners in decision making	◯	◯	◯
Item 2. Communicate the goals of the project	◯	◯	◯
Item 3. Foster respect between partners	◯	◯	◯
Item 4. Help the partners be creative and look at things differently	◯	◯	◯

Overall, how important is it that at least one "Leadership" item remains in a shortened version of the Community Engagement Survey?

◯ Least important
◯ Very important
◯ Most important

Title: "Resource Use. How well does your project use:"Response options: Response options for Resource Use items range from "not at all well" to "completely well".Domain: Section 8 of 10 of Partnership ProcessesDescription: Resource Use items "reflect partners? perceptions of how effective the project is at using the partnership?s resources and time".Instructions: Choose one importance to stakeholders response option for each item. Recall that, across this survey, you should choose about 1/3 of items to be in each importance to stakeholders response category.

	Least actionable	Very actionable: include if there is room	Most actionable: needs to be included
Item 1. The partnership's financial resources	○	○	○
Item 2. The partnership's in-kind resources	○	○	○
Item 3. The partners' time	○	○	○

Overall, how important is it that at least one "Resource Use" item remains in a shortened version of the Community Engagement Survey?

○ Least important
○ Very important
○ Most important

Title: "Trust. How much do you agree or disagree with these statements about the level of trust between partnership members?"Response options: Response options for Trust items range from "completely disagree" to "completely agree".Domain: Section 9 of 10 of Partnership ProcessesDescription: Trust items measure the partnership's "trust development" which is "dynamic" and "rests on participation (showing up), effective communication, and commitment to common goals".Instructions: Choose one importance to stakeholders response option for each item. Recall that, across this survey, you should choose about 1/3 of items to be in each importance to stakeholders response category.

	Least actionable	Very actionable: include if there is room	Most actionable: needs to be included
Item 1. I trust the decisions others make about issues that are important to our projects.	◯	◯	◯
Item 2. I can rely on the people that I work with on this project.	◯	◯	◯
Item 3. People in this partnership have a lot of confidence in one another.	◯	◯	◯

Overall, how important is it that at least one "Trust" item remains in a shortened version of the Community Engagement Survey?

◯ Least important
◯ Very important
◯ Most important

Title: "Community Engagement Principles. Does this project reflect the following Community Based Participatory Research (CBPR) principles?"Response options: Response options for Community Engagement Principles items range from "not at all" to "to a complete extent".Domain: Section 10 of 10 of Partnership ProcessesDescription: Community Engagement Principles items measure "the degree to which academic and community partners agree with principles of engagement in terms of commitment to partners, partnership, and community well-being" and "reflect how individual team members feel the research project integrates community culture(s), history and understandings in the research and intervention design and implementation".Instructions: Choose one importance to stakeholders response option for each item. Recall that, across this survey, you should choose about 1/3 of items to be in each importance to stakeholders response category.

	Least actionable	Very actionable: include if there is room	Most actionable: needs to be included
Item 1. This project builds on resources and strengths in the community.	○	○	○
Item 2. This project facilitates equitable partnerships in all phases of the research.	○	○	○
Item 3. This project helps all partners involved to grow and learn from one another.	○	○	○
Item 4. This project balances research and social action for the mutual benefit of all partners.	○	○	○
Item 5. This project emphasizes the factors that are important to the community (e.g., environmental and social factors) which affect well-being.	○	○	○
Item 6. This project communicates knowledge and findings to all partners and involves all partners in the dissemination process.	○	○	○
Item 7. This project views CBPR or community engaged research as a long term process and a long term commitment.	○	○	○
Item 8. This project is responsive to community histories.	○	○	○

Item 9. This project integrates the words and language of the community.	○	○	○
Item 10. This project connects with the ways things are done in the community.	○	○	○

Overall, how important is it that at least one "Community Engagement Principles" item remains in a shortened version of the Community Engagement Survey?

○ Least important
○ Very important
○ Most important

Title: "Community Engagement in Research Project. How much have community partners been involved in the following research steps? For steps that have not yet happened, how much will community members be involved?"Response options: Response options for Community Engagement in Research Project items range from "not at all involved" to "completely involved".Domain: Section 1 of 2 of Intervention & ResearchDescription: Community Engagement in Research Project items measure the extent to which "community members participate in all phases of the research".Instructions: Choose one importance to stakeholders response option for each item. Recall that, across this survey, you should choose about 1/3 of items to be in each importance to stakeholders response category.

	Least actionable	Very actionable: include if there is room	Most actionable: needs to be included
Item 1. Grant proposal writing	◯	◯	◯
Item 2. Background research	◯	◯	◯
Item 3. Developing sampling procedures	◯	◯	◯
Item 4. Designing and implementing the intervention	◯	◯	◯
Item 5. Designing data collection instruments (such as interviews or surveys)	◯	◯	◯
Item 6. Collecting primary data	◯	◯	◯
Item 7. Interpreting study findings	◯	◯	◯
Item 8. Writing reports and journal articles	◯	◯	◯
Item 9. Giving presentations at meetings and conferences	◯	◯	◯
Item 10. Informing the community about research progress and findings	◯	◯	◯
Item 11. Informing relevant policy makers about findings	◯	◯	◯
Item 12. Sharing findings with other communities	◯	◯	◯
Item 13. Producing useful findings for community action and benefit	◯	◯	◯

Overall, how important is it that at least one "Community Engagement in Research Project" item remains in a shortened version of the Community Engagement Survey?

◯ Least important
◯ Very important
◯ Most important

Title: "Partnership Synergy. Do you and your partners:"Response options: Response options for Partnership Synergy items range from "not at all" to "to a complete extent".Domain: Section 2 of 2 of Intervention & ResearchDescription: Partnership Synergy items measure the extent to which the partnership has the "ability to develop shared goals and strategies, recognize challenges and needs, and work together effectively".Instructions: Choose one importance to stakeholders response option for each item. Recall that, across this survey, you should choose about 1/3 of items to be in each importance to stakeholders response category.

	Least actionable	Very actionable: include if there is room	Most actionable: needs to be included
Item 1. Develop goals that are widely understood and supported in this partnership	○	○	○
Item 2. Develop strategies that are most likely to work for the community or stakeholders as a whole	○	○	○
Item 3. Recognize challenges and come up with good solutions	○	○	○
Item 4. Respond to the needs and problems of your constituency or community as a whole	○	○	○
Item 5. Work together well as a partnership	○	○	○

Overall, how important is it that at least one "Partnership Synergy" item remains in a shortened version of the Community Engagement Survey?

○ Least important
○ Very important
○ Most important

Title: "Agency Outcomes. How much do or will the community or clinical organizations in this partnership enjoy the following benefits?"Response options: Response options for Agency Outcomes items range from "not at all" to "to a complete extent".Domain: Section 1 of 6 of OutcomesDescription: Agency Outcomes items measure the extent to which the partnership has "strengthened skills of community and partner agencies to enhance their reputation, to utilize their expertise, and to affect public policy".Instructions: Choose one importance to stakeholders response option for each item. Recall that, across this survey, you should choose about 1/3 of items to be in each importance to stakeholders response category.

	Least actionable	Very actionable: include if there is room	Most actionable: needs to be included
Item 1. Enhanced reputation	○	○	○
Item 2. Enhanced ability to affect public policy	○	○	○
Item 3. Increased use of the agency's expertise or services by others	○	○	○

Overall, how important is it that at least one "Agency Outcomes" item remains in a shortened version of the Community Engagement Survey?

○ Least important
○ Very important
○ Most important

Title: "Personal Advantages. Do you or will you enjoy the following benefits from participating in this partnership?"Response options: Response options for Personal Advantages items range from "not at all" to "to a complete extent".Domain: Section 2 of 6 of OutcomesDescription: Personal Advantages items measure the extent to which individual partnership members are "feeling an enhanced sense of expertise and skills and enhanced support for more education".Instructions: Choose one importance to stakeholders response option for each item. Recall that, across this survey, you should choose about 1/3 of items to be in each importance to stakeholders response category.

	Least actionable	Very actionable: include if there is room	Most actionable: needs to be included
Item 1. Increased use of your expertise or services by others	○	○	○
Item 2. Increased ability to acquire additional financial support	○	○	○
Item 3. Increased ability to seek formal or informal education	○	○	○

Overall, how important is it that at least one "Personal Advantages" item remains in a shortened version of the Community Engagement Survey?

○ Least important
○ Very important
○ Most important

Title: "Power Relations in Research. How much do you agree or disagree that community members:"Response options: Response options for Power Relations in Research items range from "completely disagree" to "completely agree".Domain: Section 3 of 6 of OutcomesDescription: Power Relations in Research items measure the extent to which "community members feel that power is shared equally in the research process".Instructions: Choose one importance to stakeholders response option for each item. Recall that, across this survey, you should choose about 1/3 of items to be in each importance to stakeholders response category.

	Least actionable	Very actionable: include if there is room	Most actionable: needs to be included
Item 1. Have increased participation in the research process	○	○	○
Item 2. Are able to talk about the project with groups or in other settings, such as community or political meetings	○	○	○
Item 3. Can apply the findings of the research to practices and programs in the community	○	○	○
Item 4. Can voice their opinions about research in front of researchers	○	○	○
Item 5. Have the capacity or power to promote research that will benefit the community	○	○	○

Overall, how important is it that at least one "Power Relations in Research" item remains in a shortened version of the Community Engagement Survey?

○ Least important
○ Very important
○ Most important

Title: "Project Sustainability. How much do you agree or disagree that:"Response options: Response options for Project Sustainability items range from "completely disagree" to "completely agree".Domain: Section 4 of 6 of OutcomesDescription: Project Sustainability items measure the extent to which "partnership members are engaged regardless of funding and the partnership evaluates funding opportunities strategically".Instructions: Choose one importance to stakeholders response option for each item. Recall that, across this survey, you should choose about 1/3 of items to be in each importance to stakeholders response category.

	Least actionable	Very actionable: include if there is room	Most actionable: needs to be included
Item 1. I am committed to sustaining the community-academic relationship with no or low funding.	◯	◯	◯
Item 2. This project is likely to continue forward after this funding is over.	◯	◯	◯
Item 3. Our partnership carefully evaluates funding opportunities to make sure they meet both community and academic partners' needs.	◯	◯	◯

Overall, how important is it that at least one "Project Sustainability" item remains in a shortened version of the Community Engagement Survey?

◯ Least important
◯ Very important
◯ Most important

Title: "Health Outcomes"Response options: Response options for Health Outcomes items range from "not at all" to "to a complete extent".Domain: Section 5 of 6 of OutcomesDescription: Health Outcomes items measure the strength of "a partnership?s assessment that its efforts will lead to improved health in the community, along with improved health behaviors of community members".Instructions: Choose one importance to stakeholders response option for each item. Recall that, across this survey, you should choose about 1/3 of items to be in each importance to stakeholders response category.

	Least actionable	Very actionable: include if there is room	Most actionable: needs to be included
Item 1. How much do you think this project will improve the health of the community?	◯	◯	◯
Item 2. How much do you think this project will improve the health behaviors of community members?	◯	◯	◯

Overall, how important is it that at least one "Health Outcomes" item remains in a shortened version of the Community Engagement Survey?

◯ Least important
◯ Very important
◯ Most important

Title: "Future Community-Level, Research, and Policy Outcomes. How much WILL this project produce:"Response options: Response options for Future Community-Level, Research, and Policy Outcomes items range from "not at all" to "to a complete extent".Domain: Section 6 of 6 of OutcomesDescription: Future Community-Level, Research, and Policy Outcomes items measure the partnership's confidence in their ability to "reinforce cultural identity or pride, experience broad social impacts, and produce a better overall community environment", "link research efforts to community needs with an improved ability of academic partners to integrate community perspectives into research design and methods", and have efforts that "lead to policy changes".Instructions: Choose one importance to stakeholders response option for each item. Recall that, across this survey, you should choose about 1/3 of items to be in each importance to stakeholders response category.

	Least actionable	Very actionable: include if there is room	Most actionable: needs to be included
Item 1. Better coordination between agencies, researchers, and community groups	◯	◯	◯
Item 2. Changes in the nature of debates about important health issues in the community	◯	◯	◯
Item 3. Useful findings for the development of community practices, programs, or policies	◯	◯	◯
Item 4. Changes in policy	◯	◯	◯
Item 5. Changes in clinical practices	◯	◯	◯
Item 6. Better overall environment in the community	◯	◯	◯
Item 7. Reinforced cultural identity or pride	◯	◯	◯
Item 8. Broad social impacts	◯	◯	◯
Item 9. Improved academic ability to integrate community perspectives into research design and methods	◯	◯	◯
Item 10. Research better linked to community needs	◯	◯	◯

Overall, how important is it that at least one "Future Community-Level, Research, and Policy Outcomes" item remains in a shortened version of the Community Engagement Survey?

◯ Least important
◯ Very important
◯ Most important

Title: "Community Context and Capacity"Response options: Response options for Community Context and Capacity items range from "not at all" to "to a complete extent".Domain: Section 1 of 2 of ContextDescription: Community Context and Capacity items measure the extent to which "the partnership has the ability to build from community capacities and histories of advocacy to confront inequitable community conditions".Instructions: Choose one content validity response option for each item. Recall that, across this survey, you should choose about 1/3 of items to be in each content validity response category.

	Least important to content	Very important to content: include if there is room	Most important to content: needs to be included
Item 1. The community or communities participating in this project have a history of organizing services or events.	○	○	○
Item 2. The community or communities participating in this project have a history of advocating for social or health equity.	○	○	○
Item 3. By working together, people in the community or communities participating in this project have previously influenced decisions that affected their communities.	○	○	○

Overall, how important is it that at least one "Community Context and Capacity" item remains in a shortened version of the Community Engagement Survey?

- ○ Least important
- ○ Very important
- ○ Most important

Title: "Partnership Capacity"Response options: Response options for Partnership Capacity items range from "not at all" to "to a complete extent".Domain: Section 2 of 2 of ContextDescription: Partnership Capacity items measure the extent to which the partnership has "foundational resources and skills necessary for the partnership to achieve project goals".Instructions: Choose one content validity response option for each item. Recall that, across this survey, you should choose about 1/3 of items to be in each content validity response category.

	Least important to content	Very important to content: include if there is room	Most important to content: needs to be included
Item 1. Skills and expertise	○	○	○
Item 2. Diverse members	○	○	○
Item 3. Legitimacy and credibility in the community	○	○	○
Item 4. Ability to bring people together for meetings/activities	○	○	○
Item 5. Connections to relevant stakeholders	○	○	○

Overall, how important is it that at least one "Partnership Capacity" item remains in a shortened version of the Community Engagement Survey?

○ Least important
○ Very important
○ Most important

Title: "Bridging Differences"Response options: Response options for Bridging Differences items range from "not at all" to "to a complete extent".Domain: Section 1 of 10 of Partnership ProcessesDescription: Bridging Differences items measure the extent to which the partnership has "the capacity to work across difference and also includes academic team members sharing similar cultural, racial-ethnic, identity backgrounds to community partners".Instructions: Choose one content validity response option for each item. Recall that, across this survey, you should choose about 1/3 of items to be in each content validity response category.

	Least important to content	Very important to content: include if there is room	Most important to content: needs to be included
Item 1. The community partners (such as patients, community members, or organizations) have the knowledge, skills, and confidence to interact effectively with the academic partners (such as individuals from universities).	○	○	○
Item 2. The academic partners have members who are from a similar background as the community partners.	○	○	○
Item 3. The academic partners have the knowledge, skills, and confidence to interact effectively with the community partners.	○	○	○

Overall, how important is it that at least one "Bridging Differences" item remains in a shortened version of the Community Engagement Survey?

○ Least important
○ Very important
○ Most important

Title: "Mission and Strategies"Response options: Response options for Mission and Strategies items range from "completely disagree" to "completely agree".Domain: Section 2 of 10 of Partnership ProcessesDescription: Mission and Strategies items measure the extent to which the partnership has "shared values and understandings of problems, mission, priorities, and strategies".Instructions: Choose one content validity response option for each item. Recall that, across this survey, you should choose about 1/3 of items to be in each content validity response category.

	Least important to content	Very important to content: include if there is room	Most important to content: needs to be included
Item 1. Members of our partnership have a clear and shared understanding of the problems we are trying to address.	○	○	○
Item 2. Members can generally state the mission and goals of our partnership.	○	○	○
Item 3. There is general agreement with respect to the priorities of our partnership.	○	○	○
Item 4. There is general agreement on the strategies our partnership should use in pursuing its priorities.	○	○	○

Overall, how important is it that at least one "Mission and Strategies" item remains in a shortened version of the Community Engagement Survey?

○ Least important
○ Very important
○ Most important

Title: "Influence in the Partnership"Response options: Response options for Influence in the Partnership items range from "completely disagree" to "completely agree".Domain: Section 3 of 10 of Partnership ProcessesDescription: Influence in the Partnership items measure "the perception of how individual team members feel about their ability to contribute to decisions in the research team context".Instructions: Choose one content validity response option for each item. Recall that, across this survey, you should choose about 1/3 of items to be in each content validity response category.

	Least important to content	Very important to content: include if there is room	Most important to content: needs to be included
Item 1. I have influence over decisions that this partnership makes.	◯	◯	◯
Item 2. My involvement influences the partnership to be more responsive to the community.	◯	◯	◯
Item 3. I am able to influence the work on this project.	◯	◯	◯

Overall, how important is it that at least one "Influence in the Partnership" item remains in a shortened version of the Community Engagement Survey?

◯ Least important
◯ Very important
◯ Most important

Title: "Participatory Decision Making"Response options: Response options for Participatory Decision Making items range from "completely disagree" to "completely agree".Domain: Section 4 of 10 of Partnership ProcessesDescription: Participatory Decision Making items measure the extent to which the partnership has "decision making that takes all opinions into account, though there are multiple ways to achieve high levels of participation".Instructions: Choose one content validity response option for each item. Recall that, across this survey, you should choose about 1/3 of items to be in each content validity response category.

	Least important to content	Very important to content: include if there is room	Most important to content: needs to be included
Item 1. I feel comfortable with the way decisions are made in this partnership.	○	○	○
Item 2. When decisions are made, I support the decisions made by other partners in this partnership.	○	○	○
Item 3. When decisions are made, I feel that my opinion is taken into consideration by other partners in this partnership.	○	○	○

Overall, how important is it that at least one "Participatory Decision Making" item remains in a shortened version of the Community Engagement Survey?

○ Least important
○ Very important
○ Most important

Title: "Quality of Dialogue. How much do you agree or disagree that this partnership has conversations where:"Response options: Response options for Quality of Dialogue items range from "completely disagree" to "completely agree".Domain: Section 5 of 10 of Partnership ProcessesDescription: Quality of Dialogue items measure the extent to which "all partners listen and participate in dialogue with each other so that all opinions and knowledge are valued, and community members feel their voices are equally valued" and "community and academic partners interact, negotiate, and manage conflicts, tensions, and frictions that emerge in the partnered research".Instructions: Choose one content validity response option for each item. Recall that, across this survey, you should choose about 1/3 of items to be in each content validity response category.

	Least important to content	Very important to content: include if there is room	Most important to content: needs to be included
Item 1. We show positive attitudes towards one another.	◯	◯	◯
Item 2. Everyone in our partnership participates in our meetings.	◯	◯	◯
Item 3. We listen to each other.	◯	◯	◯
Item 4. When conflicts occur, we work together to resolve them.	◯	◯	◯
Item 5. Even when we don't have total agreement, we reach a kind of consensus that we all accept.	◯	◯	◯

Overall, how important is it that at least one "Quality of Dialogue" item remains in a shortened version of the Community Engagement Survey?

◯ Least important
◯ Very important
◯ Most important

Title: "Reflexivity"Response options: Response options for Reflexivity items range from "completely disagree" to "completely agree".Domain: Section 6 of 10 of Partnership ProcessesDescription: Reflexivity items measure the extent to which the partnership has the "capacity to evaluate and reflect on their own partnership processes in order to seek continual improvement; and to recognize the challenges of addressing issues of equity, power, and privilege in their research processes".Instructions: Choose one content validity response option for each item. Recall that, across this survey, you should choose about 1/3 of items to be in each content validity response category.

	Least important to content	Very important to content: include if there is room	Most important to content: needs to be included
Item 1. Our partnership has discussions about our role in promoting strategies to address social and health equity.	◯	◯	◯
Item 2. Our partnership evaluates together what we've done well and how we can improve our collaboration.	◯	◯	◯
Item 3. Our partnership reflects on issues of power and privilege within our partnership.	◯	◯	◯

Overall, how important is it that at least one "Reflexivity" item remains in a shortened version of the Community Engagement Survey?

◯ Least important
◯ Very important
◯ Most important

Title: "Leadership. How well does the leadership for the partnership:"Response options: Response options for Leadership items range from "not at all well" to "completely well".Domain: Section 7 of 10 of Partnership ProcessesDescription: Leadership items measure the extent to which the leadership for the partnership "honors knowledge and encourages participation from all partners, and supports development of community leaders as equal partners".Instructions: Choose one content validity response option for each item. Recall that, across this survey, you should choose about 1/3 of items to be in each content validity response category.

	Least important to content	Very important to content: include if there is room	Most important to content: needs to be included
Item 1. Encourage active participation of academic and community partners in decision making	◯	◯	◯
Item 2. Communicate the goals of the project	◯	◯	◯
Item 3. Foster respect between partners	◯	◯	◯
Item 4. Help the partners be creative and look at things differently	◯	◯	◯

Overall, how important is it that at least one "Leadership" item remains in a shortened version of the Community Engagement Survey?

◯ Least important
◯ Very important
◯ Most important

Title: "Resource Use. How well does your project use:"Response options: Response options for Resource Use items range from "not at all well" to "completely well".Domain: Section 8 of 10 of Partnership ProcessesDescription: Resource Use items "reflect partners? perceptions of how effective the project is at using the partnership?s resources and time".Instructions: Choose one content validity response option for each item. Recall that, across this survey, you should choose about 1/3 of items to be in each content validity response category.

	Least important to content	Very important to content: include if there is room	Most important to content: needs to be included
Item 1. The partnership's financial resources	○	○	○
Item 2. The partnership's in-kind resources	○	○	○
Item 3. The partners' time	○	○	○

Overall, how important is it that at least one "Resource Use" item remains in a shortened version of the Community Engagement Survey?

○ Least important
○ Very important
○ Most important

Title: "Trust. How much do you agree or disagree with these statements about the level of trust between partnership members?"Response options: Response options for Trust items range from "completely disagree" to "completely agree".Domain: Section 9 of 10 of Partnership ProcessesDescription: Trust items measure the partnership's "trust development" which is "dynamic" and "rests on participation (showing up), effective communication, and commitment to common goals".Instructions: Choose one content validity response option for each item. Recall that, across this survey, you should choose about 1/3 of items to be in each content validity response category.

	Least important to content	Very important to content: include if there is room	Most important to content: needs to be included
Item 1. I trust the decisions others make about issues that are important to our projects.	◯	◯	◯
Item 2. I can rely on the people that I work with on this project.	◯	◯	◯
Item 3. People in this partnership have a lot of confidence in one another.	◯	◯	◯

Overall, how important is it that at least one "Trust" item remains in a shortened version of the Community Engagement Survey?

◯ Least important
◯ Very important
◯ Most important

Title: "Community Engagement Principles. Does this project reflect the following Community Based Participatory Research (CBPR) principles?"Response options: Response options for Community Engagement Principles items range from "not at all" to "to a complete extent".Domain: Section 10 of 10 of Partnership ProcessesDescription: Community Engagement Principles items measure "the degree to which academic and community partners agree with principles of engagement in terms of commitment to partners, partnership, and community well-being" and "reflect how individual team members feel the research project integrates community culture(s), history and understandings in the research and intervention design and implementation".Instructions: Choose one content validity response option for each item. Recall that, across this survey, you should choose about 1/3 of items to be in each content validity response category.

	Least important to content	Very important to content: include if there is room	Most important to content: needs to be included
Item 1. This project builds on resources and strengths in the community.	○	○	○
Item 2. This project facilitates equitable partnerships in all phases of the research.	○	○	○
Item 3. This project helps all partners involved to grow and learn from one another.	○	○	○
Item 4. This project balances research and social action for the mutual benefit of all partners.	○	○	○
Item 5. This project emphasizes the factors that are important to the community (e.g., environmental and social factors) which affect well-being.	○	○	○
Item 6. This project communicates knowledge and findings to all partners and involves all partners in the dissemination process.	○	○	○
Item 7. This project views CBPR or community engaged research as a long term process and a long term commitment.	○	○	○

Item 8. This project is responsive to community histories.	◯	◯	◯
Item 9. This project integrates the words and language of the community.	◯	◯	◯
Item 10. This project connects with the ways things are done in the community.	◯	◯	◯

Overall, how important is it that at least one "Community Engagement Principles" item remains in a shortened version of the Community Engagement Survey?

◯ Least important
◯ Very important
◯ Most important

Title: "Community Engagement in Research Project. How much have community partners been involved in the following research steps? For steps that have not yet happened, how much will community members be involved?"Response options: Response options for Community Engagement in Research Project items range from "not at all involved" to "completely involved".Domain: Section 1 of 2 of Intervention & ResearchDescription: Community Engagement in Research Project items measure the extent to which "community members participate in all phases of the research".Instructions: Choose one content validity response option for each item. Recall that, across this survey, you should choose about 1/3 of items to be in each content validity response category.

	Least important to content	Very important to content: include if there is room	Most important to content: needs to be included
Item 1. Grant proposal writing	◯	◯	◯
Item 2. Background research	◯	◯	◯
Item 3. Developing sampling procedures	◯	◯	◯
Item 4. Designing and implementing the intervention	◯	◯	◯
Item 5. Designing data collection instruments (such as interviews or surveys)	◯	◯	◯
Item 6. Collecting primary data	◯	◯	◯
Item 7. Interpreting study findings	◯	◯	◯
Item 8. Writing reports and journal articles	◯	◯	◯
Item 9. Giving presentations at meetings and conferences	◯	◯	◯
Item 10. Informing the community about research progress and findings	◯	◯	◯
Item 11. Informing relevant policy makers about findings	◯	◯	◯
Item 12. Sharing findings with other communities	◯	◯	◯
Item 13. Producing useful findings for community action and benefit	◯	◯	◯

Overall, how important is it that at least one "Community Engagement in Research Project" item remains in a shortened version of the Community Engagement Survey?

◯ Least important
◯ Very important
◯ Most important

Title: "Partnership Synergy. Do you and your partners:"Response options: Response options for Partnership Synergy items range from "not at all" to "to a complete extent".Domain: Section 2 of 2 of Intervention & ResearchDescription: Partnership Synergy items measure the extent to which the partnership has the "ability to develop shared goals and strategies, recognize challenges and needs, and work together effectively".Instructions: Choose one content validity response option for each item. Recall that, across this survey, you should choose about 1/3 of items to be in each content validity response category.

	Least important to content	Very important to content: include if there is room	Most important to content: needs to be included
Item 1. Develop goals that are widely understood and supported in this partnership	○	○	○
Item 2. Develop strategies that are most likely to work for the community or stakeholders as a whole	○	○	○
Item 3. Recognize challenges and come up with good solutions	○	○	○
Item 4. Respond to the needs and problems of your constituency or community as a whole	○	○	○
Item 5. Work together well as a partnership	○	○	○

Overall, how important is it that at least one "Partnership Synergy" item remains in a shortened version of the Community Engagement Survey?

○ Least important
○ Very important
○ Most important

Title: "Agency Outcomes. How much do or will the community or clinical organizations in this partnership enjoy the following benefits?"Response options: Response options for Agency Outcomes items range from "not at all" to "to a complete extent".Domain: Section 1 of 6 of OutcomesDescription: Agency Outcomes items measure the extent to which the partnership has "strengthened skills of community and partner agencies to enhance their reputation, to utilize their expertise, and to affect public policy".Instructions: Choose one content validity response option for each item. Recall that, across this survey, you should choose about 1/3 of items to be in each content validity response category.

	Least important to content	Very important to content: include if there is room	Most important to content: needs to be included
Item 1. Enhanced reputation	○	○	○
Item 2. Enhanced ability to affect public policy	○	○	○
Item 3. Increased use of the agency's expertise or services by others	○	○	○

Overall, how important is it that at least one "Agency Outcomes" item remains in a shortened version of the Community Engagement Survey?

○ Least important
○ Very important
○ Most important

Title: "Personal Advantages. Do you or will you enjoy the following benefits from participating in this partnership?"Response options: Response options for Personal Advantages items range from "not at all" to "to a complete extent".Domain: Section 2 of 6 of OutcomesDescription: Personal Advantages items measure the extent to which individual partnership members are "feeling an enhanced sense of expertise and skills and enhanced support for more education".Instructions: Choose one content validity response option for each item. Recall that, across this survey, you should choose about 1/3 of items to be in each content validity response category.

	Least important to content	Very important to content: include if there is room	Most important to content: needs to be included
Item 1. Increased use of your expertise or services by others	◯	◯	◯
Item 2. Increased ability to acquire additional financial support	◯	◯	◯
Item 3. Increased ability to seek formal or informal education	◯	◯	◯

Overall, how important is it that at least one "Personal Advantages" item remains in a shortened version of the Community Engagement Survey?

◯ Least important
◯ Very important
◯ Most important

Title: "Power Relations in Research. How much do you agree or disagree that community members:"Response options: Response options for Power Relations in Research items range from "completely disagree" to "completely agree".Domain: Section 3 of 6 of OutcomesDescription: Power Relations in Research items measure the extent to which "community members feel that power is shared equally in the research process".Instructions: Choose one content validity response option for each item. Recall that, across this survey, you should choose about 1/3 of items to be in each content validity response category.

	Least important to content	Very important to content: include if there is room	Most important to content: needs to be included
Item 1. Have increased participation in the research process	◯	◯	◯
Item 2. Are able to talk about the project with groups or in other settings, such as community or political meetings	◯	◯	◯
Item 3. Can apply the findings of the research to practices and programs in the community	◯	◯	◯
Item 4. Can voice their opinions about research in front of researchers	◯	◯	◯
Item 5. Have the capacity or power to promote research that will benefit the community	◯	◯	◯

Overall, how important is it that at least one "Power Relations in Research" item remains in a shortened version of the Community Engagement Survey?

◯ Least important
◯ Very important
◯ Most important

Title: "Project Sustainability. How much do you agree or disagree that:"Response options: Response options for Project Sustainability items range from "completely disagree" to "completely agree".Domain: Section 4 of 6 of OutcomesDescription: Project Sustainability items measure the extent to which "partnership members are engaged regardless of funding and the partnership evaluates funding opportunities strategically".Instructions: Choose one content validity response option for each item. Recall that, across this survey, you should choose about 1/3 of items to be in each content validity response category.

	Least important to content	Very important to content: include if there is room	Most important to content: needs to be included
Item 1. I am committed to sustaining the community-academic relationship with no or low funding.	○	○	○
Item 2. This project is likely to continue forward after this funding is over.	○	○	○
Item 3. Our partnership carefully evaluates funding opportunities to make sure they meet both community and academic partners' needs.	○	○	○

Overall, how important is it that at least one "Project Sustainability" item remains in a shortened version of the Community Engagement Survey?

- ○ Least important
- ○ Very important
- ○ Most important

Title: "Health Outcomes"Response options: Response options for Health Outcomes items range from "not at all" to "to a complete extent".Domain: Section 5 of 6 of OutcomesDescription: Health Outcomes items measure the strength of "a partnership?s assessment that its efforts will lead to improved health in the community, along with improved health behaviors of community members".Instructions: Choose one content validity response option for each item. Recall that, across this survey, you should choose about 1/3 of items to be in each content validity response category.

	Least important to content	Very important to content: include if there is room	Most important to content: needs to be included
Item 1. How much do you think this project will improve the health of the community?	○	○	○
Item 2. How much do you think this project will improve the health behaviors of community members?	○	○	○

Overall, how important is it that at least one "Health Outcomes" item remains in a shortened version of the Community Engagement Survey?

- ○ Least important
- ○ Very important
- ○ Most important

Title: "Future Community-Level, Research, and Policy Outcomes. How much WILL this project produce:"Response options: Response options for Future Community-Level, Research, and Policy Outcomes items range from "not at all" to "to a complete extent".Domain: Section 6 of 6 of OutcomesDescription: Future Community-Level, Research, and Policy Outcomes items measure the partnership's confidence in their ability to "reinforce cultural identity or pride, experience broad social impacts, and produce a better overall community environment", "link research efforts to community needs with an improved ability of academic partners to integrate community perspectives into research design and methods", and have efforts that "lead to policy changes".Instructions: Choose one content validity response option for each item. Recall that, across this survey, you should choose about 1/3 of items to be in each content validity response category.

	Least important to content	Very important to content: include if there is room	Most important to content: needs to be included
Item 1. Better coordination between agencies, researchers, and community groups	○	○	○
Item 2. Changes in the nature of debates about important health issues in the community	○	○	○
Item 3. Useful findings for the development of community practices, programs, or policies	○	○	○
Item 4. Changes in policy	○	○	○
Item 5. Changes in clinical practices	○	○	○
Item 6. Better overall environment in the community	○	○	○
Item 7. Reinforced cultural identity or pride	○	○	○
Item 8. Broad social impacts	○	○	○
Item 9. Improved academic ability to integrate community perspectives into research design and methods	○	○	○
Item 10. Research better linked to community needs	○	○	○

Overall, how important is it that at least one "Future Community-Level, Research, and Policy Outcomes" item remains in a shortened version of the Community Engagement Survey?

○ Least important
○ Very important
○ Most important
