# Supplementary material for: A short pragmatic tool for evaluating community engagement: Partnering for Health Improvement and Research Equity
Source: Front Public Health. 2025 Jun 11;13:1539864. doi: 10.3389/fpubh.2025.1539864 (PMC12198686; doi:10.3389/fpubh.2025.1539864)
Supplement: Supplementary file 2 [file Data_Sheet_2.PDF]

Supplemental File 2: Summary Statistics for Focus Group Participants	Action-ability	Import-ance to content	Statistical import-ance	Total score out of 30
**Community Context and Capacity**				
Item 1. The community or communities participating in this project have a history of organizing services or events.	4.0	5.0	2.25	11
Item 2. The community or communities participating in this project have a history of advocating for social or health equity.	3.5	3.8	2.25	10
Item 3. By working together, people in the community or communities participating in this project have previously influenced decisions that affected their communities.	2.0	2.5	2.25	7
**Partnership Capacity**				
Item 1. Skills and expertise	5.0	4.1	3.9	13
Item 2. Diverse members	6.5	4.1	3.4	14
Item 3. Legitimacy and credibility in the community	5.8	6.5	4.8	17
Item 4. Ability to bring people together for meetings/activities	4.5	3.5	5.6	14
Item 5. Connections to relevant stakeholders	3.0	4.1	4.8	12
**Bridging Differences**				
Item 1. The community partners (such as patients, community members, or organizations) have the knowledge, skills, and confidence to interact effectively with the academic partners (such as individuals from universities).	4.2	5.0	3.5	13
Item 2. The academic partners have members who are from a similar background as the community partners.	1.0	2.1	2.0	5
Item 3. The academic partners have the knowledge, skills, and confidence to interact effectively with the community partners.	5.0	6.1	4.1	15
**Mission and Strategies**				
Item 1. Members of our partnership have a clear and shared understanding of the problems we are trying to address.	6.0	7.4	3.9	17
Item 2. Members can generally state the mission and goals of our partnership.	3.0	3.2	3.9	10
Item 3. There is general agreement with respect to the priorities of our partnership.	4.2	4.4	5.9	15
Item 4. There is general agreement on the strategies our partnership should use in pursuing its priorities.	3.5	5.3	3.9	13
**Influence in the Partnership**				
Item 1. I have influence over decisions that this partnership makes.	3.7	2.8	2.5	9

Item 2. My involvement influences the partnership to be more responsive to the community.	2.6	4.1	1.7	8
Item 3. I am able to influence the work on this project.	2.6	2.8	2.5	8
**Participatory Decision Making**				
Item 1. I feel comfortable with the way decisions are made in this partnership.	3.7	5.0	2.6	11
Item 2. When decisions are made, I support the decisions made by other partners in this partnership.	1.6	1.8	3.4	7
Item 3. When decisions are made, I feel that my opinion is taken into consideration by other partners in this partnership.	4.4	5.9	3.0	13
**Quality of Dialogue**				
Item 1. We show positive attitudes towards one another.	3.2	1.7	4.9	10
Item 2. Everyone in our partnership participates in our meetings.	2.1	2.2	2.0	6
Item 3. We listen to each other.	4.4	5.6	4.9	15
Item 4. When conflicts occur, we work together to resolve them.	5.3	5.6	3.9	15
Item 5. Even when we don't have total agreement, we reach a kind of consensus that we all accept.	3.7	5.6	3.9	13
**Reflexivity**				
Item 1. Our partnership has discussions about our role in promoting strategies to address social and health equity.	3.2	2.8	4.1	10
Item 2. Our partnership evaluates together what we've done well and how we can improve our collaboration.	6.8	6.9	3.7	17
Item 3. Our partnership reflects on issues of power and privilege within our partnership.	5.8	5.6	3.2	15
**Leadership**				
Item 1. Encourage active participation of academic and community partners in decision making	6.1	7.1	4.9	18
Item 2. Communicate the goals of the project	6.8	5.0	3.2	15
Item 3. Foster respect between partners	6.3	6.1	3.2	16
Item 4. Help the partners be creative and look at things differently	1.7	1.7	3.2	7
**Resource Use**				
Item 1. The partnership's financial resources	2.6	6.1	2.0	11
Item 2. The partnership's in-kind resources	2.6	1.8	2.0	6
Item 3. The partners' time	4.4	4.4	1.6	10
**Trust**				
Item 1. I trust the decisions others make about issues that are important to our projects.	2.6	3.3	2.5	8
Item 2. I can rely on the people that I work with on this project.	4.2	5.0	3.8	13
Item 3. People in this partnership have a lot of confidence in one another.	1.1	3.3	3.8	8
**Community Engagement Principles**				

Item 1. This project builds on resources and strengths in the community.	5.3	6.5	4.4	16
Item 2. This project facilitates equitable partnerships in all phases of the research.	4.2	6.5	5.8	17
Item 3. This project helps all partners involved to grow and learn from one another.	3.3	3.5	5.6	13
Item 4. This project balances research and social action for the mutual benefit of all partners.	3.3	3.9	4.3	12
Item 5. This project emphasizes the factors that are important to the community (e.g., environmental and social factors) which affect well-being.	3.7	5.3	3.9	13
Item 6. This project communicates knowledge and findings to all partners and involves all partners in the dissemination process.	5.3	4.4	4.3	14
Item 7. This project views CBPR or community engaged research as a long term process and a long term commitment.	3.7	5.0	3.9	13
Item 8. This project is responsive to community histories.	2.8	3.3	3.9	10
Item 9. This project integrates the words and language of the community.	3.7	5.0	3.9	13
Item 10. This project connects with the ways things are done in the community.	3.2	3.3	6.2	13
**Community Engagement in Research Project**				
Item 1. Grant proposal writing	5.3	3.8	5.0	14
Item 2. Background research	0.6	1.3	5.0	7
Item 3. Developing sampling procedures	0.6	0.0	4.6	5
Item 4. Designing and implementing the intervention	6.5	6.3	4.2	17
Item 5. Designing data collection instruments (such as interviews or surveys)	2.9	3.1	5.0	11
Item 6. Collecting primary data	2.9	2.5	2.5	8
Item 7. Interpreting study findings	6.5	6.7	6.4	20
Item 8. Writing reports and journal articles	2.4	3.8	4.9	11
Item 9. Giving presentations at meetings and conferences	4.1	3.8	5.3	13
Item 10. Informing the community about research progress and findings	8.2	8.1	5.6	22
Item 11. Informing relevant policy makers about findings	5.9	6.3	3.6	16
Item 12. Sharing findings with other communities	4.1	3.8	5.6	13
Item 13. Producing useful findings for community action and benefit	8.8	8.8	4.4	22
**Partnership Synergy**				
Item 1. Develop goals that are widely understood and supported in this partnership	5.3	6.3	2.9	14
Item 2. Develop strategies that are most likely to work for the community or stakeholders as a whole	4.1	4.4	4.0	12
Item 3. Recognize challenges and come up with good solutions	4.1	3.1	4.0	11
Item 4. Respond to the needs and problems of your constituency or community as a whole	4.1	5.7	4.3	14
Item 5. Work together well as a partnership	4.7	6.3	3.1	14

**Agency Outcomes**				
Item 1. Enhanced reputation	1.1	0.7	1.8	4
Item 2. Enhanced ability to affect public policy	2.8	5.3	1.8	10
Item 3. Increased use of the agency's expertise or services by others	3.3	1.3	3.0	8
**Personal Advantages**				
Item 1. Increased use of your expertise or services by others	2.2	2.7	2.2	7
Item 2. Increased ability to acquire additional financial support	2.8	3.3	0.9	7
Item 3. Increased ability to seek formal or informal education	1.8	0.7	0.9	3
**Power Relations in Research**				
Item 1. Have increased participation in the research process	3.3	6.0	4.1	13
Item 2. Are able to talk about the project with groups or in other settings, such as community or political meetings	3.9	4.7	5.5	14
Item 3. Can apply the findings of the research to practices and programs in the community	4.4	6.0	5.5	16
Item 4. Can voice their opinions about research in front of researchers	4.4	3.3	3.4	11
Item 5. Have the capacity or power to promote research that will benefit the community	5.9	6.7	3.4	16
**Project Sustainability**				
Item 1. I am committed to sustaining the community-academic relationship with no or low funding.	3.3	3.6	2.2	9
Item 2. This project is likely to continue forward after this funding is over.	2.8	3.3	3.8	10
Item 3. Our partnership carefully evaluates funding opportunities to make sure they meet both community and academic partners' needs.	3.3	2.7	2.7	9
**Health Outcomes**				
Item 1. How much do you think this project will improve the health of the community?	5.9	6.4	4.5	17
Item 2. How much do you think this project will improve the health behaviors of community members?	3.5	3.6	4.5	12
**Future Outcomes**				
Item 1. Better coordination between agencies, researchers, and community groups	5.6	5.3	5.2	16
Item 2. Changes in the nature of debates about important health issues in the community	1.7	2.0	5.2	9
Item 3. Useful findings for the development of community practices, programs, or policies	6.7	4.3	6.0	17
Item 4. Changes in policy	3.9	5.3	2.8	12
Item 5. Changes in clinical practices	3.9	4.7	2.6	11
Item 6. Better overall environment in the community	2.2	3.3	6.2	12
Item 7. Reinforced cultural identity or pride	2.2	2.7	4.2	9
Item 8. Broad social impacts	1.2	2.1	6.2	9

Item 9. Improved academic ability to integrate community perspectives into research design and methods	3.9	4.0	4.8	13
Item 10. Research better linked to community needs	7.8	6.7	4.8	19
