# Supplementary material for: A short pragmatic tool for evaluating community engagement: Partnering for Health Improvement and Research Equity
Source: Front Public Health. 2025 Jun 11;13:1539864. doi: 10.3389/fpubh.2025.1539864 (PMC12198686; doi:10.3389/fpubh.2025.1539864)
Supplement: Supplementary file 3 [file Data_Sheet_3.PDF]

Supplemental File 3: Guide for CBPR Content Expert and Stakeholder Focus Group

Opening, biographical, and organizational information

Thank you all so much for all the work you do to advance the practice and science of community based participatory research and community engaged research!

We value your time and expertise.

This focus group should take approximately 90 minutes depending on the time we need to discuss the questions.

This focus group is intended to help us formulate recommendations for items and scales to include in a shortened version of the Community Engagement Survey (CES).

As you remember, you participated in a survey assessing the importance to content and actionability CES items and scales. We are now ready to review draft recommendations based on our aggregated survey responses and statistical analyses of CES items and scales. In this focus group, we will be asking you for feedback on these draft recommendations using an "agree, disagree, discuss, consensus" approach.

Specifically, for each set of recommendations, we will ask you:

1) Do you agree with this recommendation? Why or why not?

2) Do you have an alternate recommendation?

3) What do you think about this alternate recommendation?

4) Thinking about this discussion, what is your consensus recommendation?

Finally, we will ask you about your overall recommendations regarding the "short form" CES.

1) Did we cut too much? Or did we cut too little?

2) Looking at the recommended items for the "short form" CES as a whole, what are your thoughts about this set of items?
